# Comparison of Strategies to Overcome Drug Resistance: Learning from Various Kingdoms

**DOI:** 10.3390/molecules23061476

**Published:** 2018-06-18

**Authors:** Hiroshi Ogawara

**Affiliations:** 1 HO Bio Institute, Yushima-2, Bunkyo-ku, Tokyo 113-0034, Japan; hogawara@sc5.so-net.ne.jp; Tel.: +81-3-3832-3474; 2 Department of Biochemistry, Meiji Pharmaceutical University, Noshio-2, Kiyose, Tokyo 204-8588, Japan

**Keywords:** drug resistance, self-resistance, phycotoxin, marine animal, terrestrial animal, plant, fungus, bacterium, antibiotic resistance

## Abstract

Drug resistance, especially antibiotic resistance, is a growing threat to human health. To overcome this problem, it is significant to know precisely the mechanisms of drug resistance and/or self-resistance in various kingdoms, from bacteria through plants to animals, once more. This review compares the molecular mechanisms of the resistance against phycotoxins, toxins from marine and terrestrial animals, plants and fungi, and antibiotics. The results reveal that each kingdom possesses the characteristic features. The main mechanisms in each kingdom are transporters/efflux pumps in phycotoxins, mutation and modification of targets and sequestration in marine and terrestrial animal toxins, ABC transporters and sequestration in plant toxins, transporters in fungal toxins, and various or mixed mechanisms in antibiotics. Antibiotic producers in particular make tremendous efforts for avoiding suicide, and are more flexible and adaptable to the changes of environments. With these features in mind, potential alternative strategies to overcome these resistance problems are discussed. This paper will provide clues for solving the issues of drug resistance.

## 1. Introduction

Antimicrobial agents, including antibiotics, once eliminated the serious infectious diseases almost completely from the Earth [1]. However, the emergence of multidrug resistant bacteria has revived bacterial pathogens once again, and has made the infectious diseases difficult-to-treat or untreatable now [2,3]. So, finding strategies for the rapidly increasing prevalence of antibiotic resistance is a major global challenge for the life science and public health sectors [4,5,6,7,8].

Living organisms produce a wide range of low molecular weight, natural organic compounds, including phycotoxins, marine toxins, toxins from terrestrial animals, phytotoxins, toxins from fungi, and antibiotics, and other kinds of bacterial toxins. These toxins have been produced and diversified evolutionally for interspecies arms races between offensive predators and defensive prey [9,10,11,12,13]. Here, predators and preys are not necessarily higher organisms but microorganisms. The producers of these toxins need to have strategies to prevent themselves from suicide [14]. These protective strategies can oscillate and evolve, depending on natural environments and the different kingdoms of the producers [15,16,17,18,19,20]. On the other hand, the nature and chemistry of the toxins themselves evolves in both offensive and defensive contexts [21,22,23,24]. It is interesting, therefore, to compare the protective and/or defensive strategies of bacteria through fungi, algae, plants, and animals. In addition, the clarification in the differences of these complex strategies may provide clues to solve the growing problems of antibiotic resistance.

## 2. Phycotoxins

Phycotoxins are toxic secondary metabolites that are produced by prokaryotic and eukaryotic algae or seaweeds [25,26,27]. These are molecules of small to medium (300~3500 Da) mass belonging to diverse groups of chemical compounds. Most phycotoxins are produced by flagellates, especially dinoflagellates. However, they are also produced by diatoms, haptophytes (prymnesiophytes) [28], raphidophytes [29], and cyanobacteria [30]. These phycotoxins are known to accumulate in seafood as a result of the marine food chain. Food poisoning in humans occurs because of the ingestion of seafood that is contaminated with these toxins. Different phycotoxins cause distinct poisoning events. These poisoning events are grouped into six classes, that is, neurotoxic shellfish poisoning (NSP), diarrheic shellfish poisoning (DSP), azaspiracid poisoning (AZP), ciguatera fish poisoning (CFP), amnesic shellfish poisoning (ASP), and paralytic shellfish poisoning (PSP) [31]. Four of the six poisonings are induced by dinoflagellate-derived polyketide toxins [32]. The two others are ASP and PSP. ASP is caused by domoic acid, a kainic acid analog that is produced by diatoms in the *Pseudo-nitschia* genus, and PSP is caused by the saxitoxins, a group of cyclic tetrahydropurine compounds that are produced by cyanobacteria, such as *Anabaena circinalis*, *Aphanizomenon* sp., and *Nostocales* sp. as well as by dinoflagellates, such as *Alexandrium catenella*, *Gymnodinium catenatum*, and *Pyrodinium bahamense* [33,34,35].

The majority of dinoflagellate toxins are polyketide in origin. Thus, polyketide compounds are discussed at first, focusing mainly on their self-resistance to phycotoxins. Polyketides are biosynthesized via the sequential condensations of small carboxylic acid subunits with an acyl starter in a fashion that is reminiscent of fatty acid biosynthesis. Both polyketide synthases (PKS) and fatty acid synthases (FAS) possess a similar set of functional domains, namely, ketoacyl synthase (KS), acyl transferase (AT), ketoacyl reductase (KR), dehydratase (DH), enoyl reductase (ER), acyl carrier protein (ACP), and thioesterase (TE). PKS are traditionally classified into three types, namely, type I, type II, and type III [36,37]. Type I PKS are large multifunctional proteins that combine several domains in one protein. Two subclasses are known for Type I PKS. Fungal iterative Type I PKS use the same set of catalytic domains on one protein several times for chain extension, analogously to vertebrate FAS. In contrast, modular Type I PKS function in a conveyor belt-like manner, in that the different catalytic domains are organized in modules comprising all of the required enzymatic functions. Each module is used only once during the polyketide assembly. Based on their size, functionalities, and complex structures, it is predicted that the dinoflagellate-derived polyketides are biosynthesized by Type I modular PKS. However, recent genome sequencing and transcriptome analysis, combined with blast analysis, indicate that monofunctional Type I PKS are present in brevetoxin-producing dinoflagellates *Karenia brevis* [38,39], *Alexandrium ostenfeldii* [40], and *Heterocapsa triqueta* [41]. In any case, these Type I PKS genes are distributed patchily in phytoplankton; they are present in *Cryptosporidium* and *Emiliania* but not in *Thalassiosira* nor in *Cyanidioschyzon* [42].

The mechanisms of polyketide biosynthesis in phycotoxins have been investigated both in eukaryotic (mainly dinoflagellates) and in prokaryotic organisms (cyanobacteria). However, only a few PKS have been analyzed at a molecular level in eukaryotic organisms and major research has been performed in prokaryotic organisms, because in eukaryotic organisms, their genome sizes range from 15 Gbp to 150 Gbp [43]; chromosome copy numbers vary markedly from 4 to 220 [44]; and genomes are very complex as a result of gene duplication, lateral gene transfer, endosymbiotic gene transfer events [45], and so on. Cylindrospermopsin is produced by cyanobacterial species such as *Cylindrospermopsis raciborskii*, *Aphanizomenon ovalisporum*, *Umezakia natans*, *Raphidiopsis curvata*, and *Anabaena bergii*. It has hepatoxic and neurotoxic effects and is a potential carcinogen. Its toxicity is due to the inhibition of glutathione and protein synthesis, as well as the inhibition of cytochrome P450. The toxin is a polyketide-derived alkaloid with a central functional guanidine moiety and a hydroxymethyluracil. Feeding experiments with isotope-labeled precursors have shown that guanidinoacetate is the starter unit for cylindrospermopsin biosynthesis, and successive additions of five intact acetate units onto guanidinoacetate yield the carbon backbone of cylindrospermopsin [46]. Afterward, cylindrospermopsin biosynthetic gene clusters were cloned from three producing species, *Cylindrospermopsis raciborskii* AWT205 (43 kb), *Aphanizomenon* sp. strain 10E6 (57 kb), and *Oscillatoria* sp. Strain PCC 6506 (45 kb) [47,48,49] (GenBank accession Nos. EU140798, GQ385961, and FJ418586; GB No. hereafter). A comparison of these gene clusters indicates that they are homologous and evolutionarily related, and are diverged from a common ancestor, but a substantial shuffling occurred in these organisms. It is interesting that the multidrug exporter gene, *cyrK* (GB No. ABX60156), exists within the gene clusters, indicating that it functions as a strategy of self-resistance against cylindrospermopsin.

Jamaicamide A is produced by filamentous cyanobacterium, *Lyngbya majuscula*. It is a highly functionalized lipopeptide and shows sodium channel blocking activity. Feeding experiments with labeled precursor have mapped out series of acetate and amino acid residues on the structure. The major metabolic pathway employs two modular biosynthetic systems, nonribosomal peptide synthetases (NRPS), which are responsible for assembling amino acids; and polyketide synthases (PKS), for linking together acetate as the primary building block. Edwards et al. cloned the jamaicamide-producing gene cluster as a 58 kb DNA fragment composed of 17 open reading frames [50] (GB No. AY522504). They show exact collinearity with their expected utilization, form the operon *jamABCDEFGHIJKLMNOP*, and are transcribed in the same direction, except for the last gene, *jamQ*. The last ORF (Open reading frame) JamQ, which is thought to be involved in the cyclization of the pyrrolinone ring of the molecule, is transcribed in the reverse direction. The gene cluster is preceded by a long untranslated leader region (at least 844 bp), but its exact function is not clear yet [50,51]. No resistance-related gene has been found within the gene cluster.

Hectochlorin was also isolated from *Lyngbya majuscula*. It is a cyclic lipopeptide and exhibits antifungal activity against *Candida albicans* and antiproliferative activity because of the stimulation of actin assembly [52]. The structure of hectochlorin indicates that it is derived from a mixed PKS/NRPS pathway. The cloning of the biosynthetic gene cluster supports this suggestion [53]. It consists of eight open reading frames spanning 38 kb (GB No. AY974560). All of the eight genes are transcribed in the same direction. However, no resistance-related gene has been found within the gene cluster. 

Curacin A was also obtained from *Lyngbya majuscula*. It has a unique structure containing the sequential positioning of a thiazoline and cyclopropyl ring that have been biosynthesized through the PKS/NRPS pathways. It is a cancer cell toxin as a result of the blocking of the cell cycle progression, by interacting with the colchicine binding site on tubulin and inhibiting microtubule polymerization. The biosynthetic gene cluster was cloned as a 64 kb DNA fragment and the metabolic system shows a very high level of collinearity between the genes in the cluster and the predicted biochemical steps [54,55] (GB Nos. AY652953 and HQ696500). All of the 14 genes are transcribed in the same direction. However, no resistance-related gene has been found within the cluster. As some ABC type transporter genes are found in the *Lyngbya majuscula* 3L genome [55], these transporters may be involved in the excretion of the toxins, similar to the case of cylindrospermopsin (GB No. GL890825). Amphidinolides and amphidinols from the genus *Amphidinium* dinoflagellates have similar structures to curacins [32,56].

Apratoxin A was isolated from *Lyngbya* (*Moorea*) *bouillonii* and has a structure that is composed of a polyketide section that is fused with a modified pentapetide to form a cyclic lipopeptide. Apratoxin A inhibits signal transducer and activator of transcription (STAT) 3 phosphorylation in various cell types, and induces pronounced G_1_ cell cycle arrest and apoptosis [57]. The cloned 58 kb biosynthetic gene cluster is composed of 12 open reading frames and has a Type I modular mixed PKS-NRPS organization [58]. No resistance-related gene has been found within the gene cluster. However, adjacent to the polyketide synthase genes, many ABC transporter genes are present, indicating that these ABC transporters may excrete the toxin from the cells for self-resistance (For example: GB Nos. OLT63032 and WP_075905632).

Lyngbyatoxin is an indole alkaloid first identified from a *Moorea producens* bloom having a tumor promoter activity due to the activation of protein kinase C. The 11.3 kb biosynthetic gene cluster contains four open reading frames encoding a bimodular nonribosomal peptide synthetase, cytochrome P450 monooxygenase, and a protein that is related to an oxidase/reductase [59] (GB No. AY588942). All of these fragments are transcribed in the same direction. The entire lyngbyatoxin gene cluster was attempted, in order to express heterologously in *Streptomyces coelicolor* A3(2) and *Anabaena* sp. strain PCC7120 [60,61]. Although the expression of the entire gene clusters were unsuccessful, cytochrome P45, monooxygenase LtxB, and the reverse prenyltransferase LtxC genes were accomplished, so as to express in *S. coelicolor* A3(2). In this gene cluster, no transporter nor resistance-related gene has been identified.

Teleocidin B, a protein kinase C activator that is produced by *Streptomyces blastmyceticus* NBRC 12747, is an analogue of lyngbyatoxin. The biosynthetic gene cluster of teleocidin B, composed of 23.2 kb of DNA fragments, was cloned and sequenced [62] (GB No. AB937114). It contains 15 open reading frames, including *tleA* for a nonribosomal peptide synthetase, *tleB* for a P-450 monooxygenase, *tleC* for an aromatic prenyltransferase, and three genes for ABC transporter. Interestingly, *Streptomyces lividans* TK21, containing the cluster, produces lyngbyatoxin but not teleocidin B. The essential gene for the biosynthesis of teleocidin B, *tleD*, is located outside of the *tle* cluster (GB No. AB937726). Furthermore, three ABC transporter genes are present, adjacent to the *tleABC* genes. These ABC transporters may excrete teleocidin B as well as lyngbyatoxin from the cells. 

Hepatotoxic microcystins are a family of heptapeptides that are produced by bloom-forming freshwater cyanobacteria, such as *Mycrocystis*, *Planktothrix*, and *Anabaena* [63]. The microcystins contain a number of unusual amino acid residues, including 3-amino-9-methoxy-2,6,8-trimethyl-10-phenyl-4,6-decadieniic acid, 3-methylaspartic acid, and *N*-methyl-dehydroalanine. The closely related pentapeptide nodularin is found frequently in the cyanobacteria of the species of *Nodularia spumigena* [64]. Microcystins and nodularin inhibit eukaryotic protein phosphatases of Type 1 and Type 2a, and are able to penetrate the liver cells via active transport. Biochemical and genetic studies, including feeding experiments with labelled precursors, suggest that the microcystins are biosynthesized by a mixed PKS/NRPS pathway. The biosynthetic gene cluster for microcystin spanning 55 kb was cloned (GB No. AF183408). It is composed of 10 bidirectionally transcribed open reading frames that are arranged in two putative operons, *mcyA-mcyC* and *mcyD-mcyJ*. The *mcyD-mcyJ* gene cluster contains seven open reading frames, all of which are transcribed in the opposite direction to the putative *mcyABC* operon. Among them, the 1617 bp open reading frame *mcyH* encodes a putative 37,000 Da transmembrane protein, belonging to the ABC transporter. Although no obvious function can be assigned to McyH, it is possible to speculate that McyH may play a role in the excretion of the toxin [65,66]. This speculation is supported by phylogenetic analysis [67]. Additionally, it was reported that cyanobacterial phosphoprotein phosphatase (PPP) family protein phosphatases, such as PP1-cyano1 and PP1-cyano2 from *Microcystis aeruginosa* PCC 7820, are resistant to microcystin-LR [68]. Thus, the targets are also resistant to microcystin-LR in the producer organism. 

The microcystin-related cyclic pentapeptide, nodularin, is produced by *Nodularia spumigena*. The 48 kb gene cluster of nodularin consists of nine open reading frames, *ndaA* to *ndaI* (GB No. AY210783). Similar to the case of microcystin, they are transcribed from a bidirectional regulatory promoter region and encode the nonribosomal peptide synthetase modules, polyketide synthase modules, and tailoring enzymes. NdaI consisting of 601 amino acid residues is an ABC transporter. The comparison of the gene clusters for microcystin and nodularin and of the condensation domains of NdaA and McyA/McyB revealed that extensive gene arrangements occurred between the two clusters, and that the gene cluster of nodularin evolved from a microcystin synthetase progenitor [64].

Aplysiatoxin, isolated from sea hare *Stylocheilus longicauda* and cyanobacteria, such as *Lyngbya majuscula*, *Schizothrix calcicol*a, and *Trichodesmium erythraeum*, is composed of a 14-membered bis-macrocyclic ring and a side chain containing an aromatic ring. Like lyngbyatoxin, aplysiatoxin induces dermatitis through the activation of protein kinase C. It is also a tumor promoter. Nhatrangin, possessing many structural similarities to aplysiatoxin, is suggested to be putative starter units for the aplysiatoxin biosynthetic pathway [69]. However, the biosynthetic gene clusters of these metabolites have not been cloned. 

Saxitoxins are the most renowned molecules, known as the paralytic shellfish toxins. However, they are originally biosynthesized by cyanobacteria, such as *Anabaena circinales*, *Aphanizomenon grazile*, *Cylindrospermopsis raciborskii*, and *Lyngbya wollei*, and by eukaryotic dinoflagellates, such as *Alexandrium*, *Gymnodinium*, and *Pyrodinium* [34,70], and, subsequently, are transferred to various invertebrate and vertebrate species through the aquatic freshwater and marine food chains. Saxitoxins are a group of carbamate alkaloid toxins consisting of a tetrahydropurine group and two guanidinium moieties. Intoxication with saxitoxins in humans may result in the severe and occasionally fatal illness known as paralytic shellfish poisoning. This illness is caused by the binding of saxitoxins to the α-subunit of voltage-gated Na^+^ channels (Nav). This is mediated by the interaction between the positively charged guanidinium groups of saxitoxins, with the negatively charged carboxyl groups at site 1 of the Na^+^ channel, thereby obstructing the entry of sodium ions through the pore and blocking nerve and muscle action potentials [71]. Interestingly, softshell clams (*Mya arenaria*) from areas that are exposed to red tides are more resistant to saxitoxins and accumulate saxitoxins at greater rates than the sensitive clams from unexposed areas. The resistance in the clams to saxitoxins is caused by a natural mutation of only one amino acid residue, which causes a 1000-fold decrease in affinity at the saxitoxin-binding site in the sodium channel pore. Thus, paralytic shellfish toxins like saxitoxins may act as natural selection agents, leading to a greater toxin resistance in the clam populations and an increased risk of paralytic shellfish poisoning to humans [72,73].

Saxitoxins are the most renowned molecules, known as the paralytic shellfish toxins. However, they are originally biosynthesized by cyanobacteria, such as *Anabaena circinales*, *Aphanizomenon grazile*, *Cylindrospermopsis raciborskii*, and *Lyngbya wollei*, and by eukaryotic dinoflagellates, such as *Alexandrium*, *Gymnodinium*, and *Pyrodinium* [34,70], and, subsequently, are transferred to various invertebrate and vertebrate species through the aquatic freshwater and marine food chains. Saxitoxins are a group of carbamate alkaloid toxins consisting of a tetrahydropurine group and two guanidinium moieties. Intoxication with saxitoxins in humans may result in the severe and occasionally fatal illness known as paralytic shellfish poisoning. This illness is caused by the binding of saxitoxins to the α-subunit of voltage-gated Na^+^ channels (Nav). This is mediated by the interaction between the positively charged guanidinium groups of saxitoxins, with the negatively charged carboxyl groups at site 1 of the Na^+^ channel, thereby obstructing the entry of sodium ions through the pore and blocking nerve and muscle action potentials [71]. Interestingly, softshell clams (*Mya arenaria*) from areas that are exposed to red tides are more resistant to saxitoxins and accumulate saxitoxins at greater rates than the sensitive clams from unexposed areas. The resistance in the clams to saxitoxins is caused by a natural mutation of only one amino acid residue, which causes a 1000-fold decrease in affinity at the saxitoxin-binding site in the sodium channel pore. Thus, paralytic shellfish toxins like saxitoxins may act as natural selection agents, leading to a greater toxin resistance in the clam populations and an increased risk of paralytic shellfish poisoning to humans [72,73].

The biosynthetic gene clusters of saxitoxins were cloned from cyanobacteria [34,74,75,76,77], and dinoflagellates [78]. The comparative analysis of the saxitoxin gene clusters in five species of cyanobacteria, that is, *Cylindrospermopsis raciborskii* T3 (GB No. DQ787200), *Anabaena circinalis* AWQC131C (GB No. DQ787201), *Aphanizomenon* sp. NH5 (GB No. EU603710), *Lyngbya wollei* (GB No. EU603711), and *Raphidiopsis brookii* D9 (GB No. ACYB00000000), indicates that the extensive shuffling of the genes that are involved in the biosynthesis of saxitoxins occurred among these species. Saxitoxins may be excreted through SxtF and SxtM, two multidrug and toxic compound extrusion (MATE) family transporters. Intriguingly, *sxtM* is present in all five *sxt* gene clusters, but *sxtF* is only present in *C. raciborskii* T3 and *R*. *brookii* D9. The two domains thata are involved in Na^+^ and drug recognition from NorM proteins (MATE family proteins [79]) of *Vibrio parahaemolyticus* and *V. cholera* are present in SxtF and SxtM [79]. In *L*. *wollei*, three *sxtM* genes are present. Therefore, these exporters may function in the resistant mechanisms of saxitoxin-producing bacteria and/or Nav themselves in the toxin producers that are resistant to saxitoxins or the mutations of the Nav result in the resistance. It is known that the structures of Nav is completely different in bacteria from those in eukaryotic organisms [80,81,82]. In two saxitoxin-producing dinoflagellate strains, *Alexandrium fundyense* CCMP1719 and *A. minutum* CCMP113, the analysis of *sxtA*, the starting gene of saxitoxin synthesis, showed that the dinoflagellate transcripts of *sxtA* have the same domain structure as the cyanobacterial *sxtA* genes, but the dinoflagellate transcripts are monocistronic, have a higher GC content, and contain typical dinoflagellate spliced-leader sequences and eukaryotic polyA-tails. Interestingly, in these eukaryotic dinoflagellate strains, two transporter genes *sxtF* and *sxtM,* were conserved [78].

The *sxtA* encodes a polyketide synthase in saxitoxin-producing cyanobacterium *Anabaena circinalis*. It is interesting evolutionally that SxtA is comprised of two distinct regions, namely, the N-terminal region of about 800 amino acids and the C-terminal region of about 390 amino acids; the former contains an acyl-CoA *N*-acyltransferase and a phosphopantetheine binding domain, which are homologous to those from proteobacteria, such as *Myxococcus xanthus* and *Burkholderia ambifaria;* and the latter shares a significant identity to a class I and II aminotransferase from actinobacteria, such as *Frankia alni* and *Catenulispora acidiphila*. In dinoflagellate *Alexandrium tamarense*, SxtA is split into two proteins corresponding to the N-terminal portion containing the methyltransferase and acyl carrier protein domains, and a C-terminal portion with the aminotransferase domain. The evolutional relationships of the saxitoxin biosynthetic genes in cyanobacteria and dinoflagellates were also analyzed [83,84,85,86]. 

Anatoxin A is a neurotoxic alkaloid and an agonist of the nicotinic acetylcholine receptor. Anatoxin A induces a neuromuscular blockade, resulting from muscle membrane depolarization and desensitization; impairs blood pressure, heart rate, and gas exchange causing hypoxia, muscle spasm, paralysis, and respiratory arrest; and finally death [87]. The gene cluster responsible for the biosynthesis of anatoxin A was identified in *Oscillatoria* sp., *Anabaena flos-aquae* 37, and *Cylindrospermum stagnale* PCC7417 [88,89,90]. The gene clusters from *Oscillatoria* sp. PCC6506 and *Oscillatoria* sp. PCC 6407 are identical, and those from *Anabaena flos-aquae* 37 and *Cylindrospermum stagnale* PCC7417 are similar to that of *Oscillatoria* sp. PCC6506, but they are slightly rearranged. The clusters contain three polyketide synthase genes, one acyl carrier protein gene, and one transporter gene. On the basis of the clusters, the biosynthetic route of anatoxin A was proposed [89]. The transporter AnaI may be responsible for the excretion of anatoxin A from the cells.

The hapalindole-type family of natural products is a group of lipophilic indole alkaloids that are produced by members of the cyanobacterial species of the order *Stigonematales*. This family includes hapalindoles, fisherindoles, ambiguines, and welwitindolinones [91]. These alkaloids show insecticidal, fungicidal, phytotoxic, and antialgal properties. Welwitindolinone A isonitrile shows antibacterial, antifungal, and antimycobacterial activities, and hapalindole A, fisherindole L, and *N*-methyl-welwitindolinone C isothiocyanate display cytotoxic activity against various cancer cells [92]. Interestingly, *N*-methyl-welwitindolinone C isothiocyanate attenuates the resistance of human breast carcinoma MCF-7/ADR cells to anticancer drugs, including vinblastine, taxol, actinomycin D, daunomycin, and colchicine, without affecting the cytotoxicity of cisplatin [93].

The biosynthetic gene clusters of the hapalindole-type alkaloids were cloned from the cyanobacterial strains *Fischerella* sp. ATCC 43239, *Fischerella* sp. PCC 9339, *Fischerella ambigua* UTEX 1903, *Hapalosiphon welwitschii* UH IC-52-3, *Hapalosiphon welwitschii* UTEX B1830, and *Westiella intricate* UH HT-29-1 [94,95,96]. There are three drug efflux pump or ABC transporter genes in the hapalindole gene cluster of *Fischerella* sp. PCC 9339 [IMG Gene IDs: 2517064622, 2517064623 and 2517064634], in the fisherindole gene cluster of *Fischerella muscicola* UTEX 1829 (GB Nos. APZ79543, APZ79544 and APZ79545), and in the ambiguine gene cluster of *Fischerella ambigua* UTEX 1903 (GB Nos. KJ742065 and KF664586). In the welwitindolinone biosynthetic gene cluster, on the other hand, there is one multidrug transporter of 105 amino acid residues of EmrE family in *Westiella intricata* UH HT-29-1 (GB No. AIH14815) and in *Hapalosiphon welwitschii* UH IC-52-3 (GB No. AIH14769). These transporters and/or efflux pumps may play an important role in the exclusion of hapalindole-type alkaloids. 

Ciguatera fish poisoning is a food-borne disease that is endemic to tropical and subtropical coral reef regions of the world. However, as a result of the recent global warming, international trade, and increased nutrient loading, ciguatera is now emerging as a significant issue in Asia, America, and Europe [97]. The ciguatera fish poisoning is caused by the consumption of fish that are contaminated with ciguatoxins. Ciguatoxins are produced by benthic dinoflagellates of the genus *Gambierdiscus* and are concentrated in commonly consumed fish in the tropical and subtropical regions of the world, through the marine food chain. They are heat-stable, lipophilic polycyclic ethers of complex structures, and their molecular weights are 1000~1500 Da. The pharmacology of ciguatoxins is characterized by their ability to cause the persistent activation of Nav, to increase neuronal excitability and neurotransmitter release, and to cause cell swelling, leading to a complex array of gastrointestinal, neurological, and cardiovascular symptoms [98,99].

Remarkable structural similarities between polyether ladder toxins, like ciguatoxins, brevetoxins, maitotoxin, yessotoxin, okadaic acid, and gambierol, which are derived from the marine eukaryotes dinoflagellates, and monensin, a polyether-type antibiotic that is isolated from *Streptomyces cinnamonensis*, suggest that these toxins are biosynthesized through the polyketide route in a manner that is analogous to that of monensin assembly [31,32,100,101,102]. Monensin is shown to be biosynthesized by the modular type I PKS genes [103,104]. This is confirmed by the isotope incorporation experiments. However, the detailed biosynthetic mechanisms of ciguatoxins have not been explored at the genetic level, although similar biosynthetic pathways may also be employed in dinoflagellates. Through this connection, Monroe and Van Dolah [38] identified eight polyketide synthase transcripts in brevetoxin-producing *Karenia brevis*, by a high throughput cDNA library screening. Although there is no direct linking of these transcripts to brevetoxin biosynthesis, some transcripts contain polyadenylation, 3′-untranslated regions (UTRs), and an identical dinoflagellate-specific spliced leader domains at the 5′ end of PKS synthase transcripts. In addition, Kohli et al. [105] reported two gene clusters that were unique to maitotoxin-producing dinoflagellate species *Gambierdiscus australes* and *G. belizeanus*, suggesting that these clusters may be associated with maitotoxin biosynthesis. However, no transporter-related gene has been described. Other polyether ladder toxins are palytoxin [106] from dinoflagellates, soft corals, and cyanobacteria; and ostreocin from dinoflagellate *Ostreopsis siamensis* [107]. Predators such as a starfish (*Acanthaster planci*) and fish (*Chaetodon* species) feed on the *Palythoa* colonies and accumulate high toxin concentrations in their organs in its active form. The predators can tolerate high toxin concentrations by sequestration [108]. However, the biosynthetic genes have not been cloned. Okadaic acid, a cytotoxic polyether, is biosynthesized by marine dinoflagellates of the genus *Prorocentrum* and is a causative toxin of diarrhetic shellfish poisoning. It is an inhibitor of the eukaryotic serine/threonine protein phosphatase Type 1 and 2a, and is a promotor of tumors [100]. Interestingly, non-toxic sulfated diesters of okadaic acid and dinophysis toxin DTX-1, a derivative of okadaic acid, are initially biosynthesized in the dinoflagellate cells, indicating that these sulfated diesters make the producer resistant to okadaic acid [109].

Domoic acid is a neurotoxin and is biosynthesized by the marine diatom *Pseudo-nitzschia australis*, and related species [110]. The toxin targets ionotropic glutamate receptors that are present in various vital organs, inducing memory impairment, coma, recurrent seizures, and epilepsy. Kainic acid isolated from the red alga *Digenia simplex* and acromelic acid derived from the toxic fungus *Paralepistopsis acromelalga* are analogues of domoic acid [111,112,113]. To date, the domoic acid biosynthetic genes and the biosynthetic reactions have not been described. Examining the labeling patterns of domoic acid tht is produced in *Pseudo-nitzschia* cultures, it was proposed that domoic acid arises from the condensation of the C10 isoprenoid with glutamic acid, an activated C5 product of the TCA cycle [114,115]. In addition, Boissonneault et al. [116] identified some genes that were up-regulated under domoic acid-producing conditions, using microarray and RT-qPCR methods. These include a cycloisomerase, an SLC6 transporter [117], phosphoenolpyruvate carboxykinase, glutamate dehydrogenase, a small heat shock protein, and an aldo-keto reductase. Interestingly, the cycloisomerase, the SLC6 transporter, and the aldo-keto reductase genes had a statistically significant increase in accord with the increase in the domoic acid production. Thus, the SLC6 transporter may play an important role in the movement of domoic acid, into or out of cells.

Cyanobactins are defined as ribosomally synthesized peptides with post-translational modifications, which are produced by cyanobacteria [118,119]. Previously, they were thought to be biosynthesized in the tunicate *Lissoclinum patella*. It is now demonstrated that the cyanobacterium, *Prochloron*, a symbiont of the tunicate, is in fact responsible for the production of cyanobactins, through a post-ribosomal peptide synthesis pathway.

Patellamides are members of cyanobactin-group compounds. They are cytotoxic cyclic peptides and have reverse multidrug resistance in human cancer cells [120,121,122]. In 2005, Schmidt et al. cloned a 11 kb DNA fragment comprising *patA*-*patG* genes, which are responsible for the biosynthesis of patellamide A and patellamide C [123] (GB No. AY986476). The *patE* gene encodes a patellamide A and C precursor peptide of 71 amino acid residues, the first 37 of which serve as a leader sequence for processing. Of the remaining 34 amino acid residues, 16 amino acids constitute directly the patellamide A and patellamide C sequences, whereas the remaining 18 amino acids make up the motifs directing the cyclization of patellamides. Other gene products, such as PatA, PatD, and PatG, may be involved in the post-translational modification, leading to the biosynthesis of patellamide A and patellamide C [124,125]. ABC transporters are found in these gene clusters, indicating that these transporters may function as an excretion of these toxic substances from the cells [118]. Similar type of gene clusters are found in biosyntheses of microcins that are produced by Gram-negative bacteria [126], bacteriocins in Gram-positive bacteria [127], microviridin in cyanobacteria [128], and goadsporin in *Streptomyces* [129].

Microcins are gene-encoded antimicrobial peptides that are produced by Gram-negative bacteria, especially Enterobacteria [130]. They belong to a large family of bacteriocins and are involved in microbial competition. Recently, the complete genome sequence of a microcin B-producing *Pseudomonas antarctica*, PAMC 27494, was determined [131] (GB No. CP015600). The microcin B precursor that is encoded by mcbA is post-translationally processed to the mature form by McbBCD. The *mcbE* and *mcbF* genes encode the microcin ABC transporter system (GB Nos. ANF87043 and ANF87042), indicating that the processed microcin B is exported through this system outside of the cells. Another microcin-group antibiotic microcin C7 acts as a bactericide by inhibiting the aspartyl-tRNA synthetase and stalling the protein translation machinery. The biosynthetic gene cluster for microcin C7 on a plasmid was cloned and sequenced [132] (GB No. X57583). The cloned biosynthetic gene cluster consists of six open reading frames, namely, mccA, mccB, mccC, mccD, mccE, and mccF. The 21 bp *mccA* gene encodes the heptapeptide precursor, and mccC and mccE encode an efflux pump and acetyltransferase, respectively. Thus, at least two proteins, MccC and MccE, are implicated in the self-resistance of the producing strains to microcin C. Furthermore, MccF also involves in self-immunity [133,134].

The biosynthetic gene cluster of goadsporin was cloned from *Streptomyces* sp. TP-A0584 [129] (GB No. AB205012). The cluster contains a structural gene, godA, and nine *god* genes that are implicated in post-translation modification, immunity, and transcriptional regulation. GodB and GodC show a sequence similarity to the members of the ABC transporter family and may be responsible for the translocation of goadsporin to the cell membrane, and the excretion of goadsporin to outside of the producing cells [135]. Table 1 shows the resistance-related genes in the biosynthetic gene clusters of phycotoxins and related compounds. Summarizing these results, it is apparent that transporters, exporters, and efflux pumps play a major role in the self-resistance against phycotoxins in the producer organisms. Although the modification of toxins is also observed, like okadaic acid, it is only a rare case.

## 3. Marine Toxins

**Figure 1:**
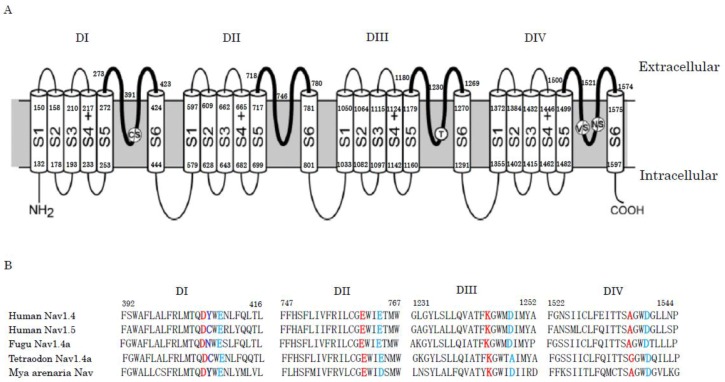
(**A**) Construction of human voltage-gated sodium channel 1.4. (**B**) Comparison of amino acid residues of human Nav1.4, human Nav1.5, Fugu Nav1.4a, *Tetraodon* Nav1.4a, and *Mya arenaria* Nav. The amino acid numbering is according to that of human Nav1.4. The GenBank accession numbers are human Nav1.4: P35499; human Nav1.5: Q14524; fugu Nav1.4a: ABB29441; Tetraodon Nav1.4a: ABB29443; and Mya arenaria Nav: AAX14719. The amino acids in alanine (A) in DIV (DEKA) are marked with red, those in two aspartate residues in DIII and DIV (EEDD) are marked with light blue, and the amino acid residues at 407 are marked with dark blue.

Electrical signaling across lipid membranes is essential for communication within and between cells. Ion-channels can pass the rapid and selective movements of one or several species of ions across the cell membrane. Voltage-gated ion channels are activated by changes in the local membrane potential. Voltage-gated sodium channels (Nav) play an essential role in the initiation and propagation of action potentials in neurons and other electrically excitable cells, such as myocytes and endocrine cells [71,136,137,138]. The Nav of human and mouse consist of α-subunit of 260 kDa and β-subunit of 30~40 kDa. Among them, the α-subunit is sufficient for functional Nav. The α-subunits of Nav are encoded by 10 genes, which are expressed in different excitable tissues [139]. Nav1.1, Nav1.2, Nav1.3, and Nav1.6 are the primary Nav in the central nervous system; Nav1.7, Nav1.8, and Nav1.9 are the primary Nav in the peripheral nervous system; Nav1.4 is the primary Nav in skeletal muscle; and Nav1.5 is the primary Nav in heart. The 10th sodium channel protein is not voltage-gated. Nav1.1, Nav1.2, Nav1.3, Nav1.4, Nav1.6, and Nav1.7 are tetrodotoxin-sensitive, and their IC_50_ are less than 10 nM. Nav1.5, Nav1.8, and Nav1.9 are tetrodotoxin-insensitive, and their IC_50_ are 1~10 μM. The α-subunits are large, single-chain polypeptides that are organized in four homologous domains, designated DI to DIV. Each domain consists of six trans-membrane helical segments, named S1 to S6. Segments S1 to S4 from each domain form the voltage-sensing domain (VSD). The four voltage-sensing domains are arranged around a central aqueous channel that is formed by the pore domain (PD). The pore domain (PD) includes the selectivity filter (SF). The selectivity filter (SF) is composed of aspartate (D) in DI, glutamate (E) in DII, lysine (K) in DIII, and alanine (A) in DIV (DEKA). The ring playing an important role in Na^+^ permeation is composed of two glutamates in DI and DII and two aspartate residues in DIII and DIV (EEDD). These amino acid residues are located just three residues downstream from those in the DEKA ring (Figure 1).

Tetrodotoxin is a deadly neurotoxin that selectively blocks Nav. Although tetrodotoxin is popularly known in Japan as the toxin in pufferfish, it is present in a diverse group of animals, including gobies, newts, frogs, horseshoe crabs, blur-ringed octopus, starfish, and red alga, dinoflagellates, and bacteria [140,141,142]. A general hypothesis is that a symbiotic or commensal bacterium living within these organisms is responsible for tetrodotoxin production. This hypothesis is supported by the fact that when pufferfish were fed a tetrodotoxin-free diet in an environment, they became nontoxic [143]. Interestingly, a comparison of the protein sequences of the skeletal muscle Nav shows that the tyrosine residue (Y407) in the pore loop of DI is substituted by the non-aromatic amino acid residue asparagine (N) or cysteine (C) in the tetrodotoxin-resistant fugu and *Tetraodon* channels (Figure 1). Furthermore, in the tetrodotoxin-insensitive human Nav1.5 channel from the heart muscle, it is replaced by cysteine (C). Some garter snake populations from different geographical locations are resistant to tetrodotoxin, however they conserve the aromatic amino acid residue (Y) at position 407. Instead, substitutions of several amino acids in the pore loop of DIV are responsible for tetrodotoxin resistance. Thus, tetrodotoxin attains a defensive role that protects the prey species from predation. However, some predators, like snakes, prey on tetrodotoxin-bearing animals, such as newts [10,11,144,145,146,147,148]. As described above, the saxitoxins bind to the same amino acid residues in the pore loop region on Nav as the tetrodotoxin. Neuronal Nav from the saxitoxin-resistant softshell clams (*Mya arenaria)* have the aromatic amino acid residue at position 407 intact, but glutamate (E) at position 764 in the pore loop of DII is substituted by aspartate (D) [72,73,149]. 

Tetrodotoxin is known to be biosynthesized by various bacteria, including actinobacteria, bacteroides, firmicutes, and proteobacteria [140,141]. However, its biosynthetic mechanism has not been clarified. Therefore, the self-resistance mechanism in these microorganisms remains to be defined. Tarichatoxin that has been isolated from the *Taricha* newts of California [150] and maculotoxin that has been isolated from *Hapalochlaena maculosa* (the blue-ringed octopus; [151]) have the same chemical structures as tetrodotoxin.

Cone snails, which are predatory marine gastropods feeding on fish, worms, or snails, produce a cocktail of venoms that are used for predation, defense, and competition. The major venom components are conotoxins or conopeptides. They are remarkably diverse in terms of structure and function [152,153,154]. Over 10,000 conotoxins or conopeptides are identified. They are biosynthesized as propeptides and are subject to extensive post-translational modifications in order to form mature peptides. The propeptides are cleaved by specialized venom endopeptidases belonging to the pathogenesis-related protein superfamily [155]. The mature peptides are comprised of 12~50 amino acid residues and 1~5 disulfide bridges [156]. Once they are injected into the prey or predators (fish, molluscs, or worms) [157], they act as fast-acting paralytics [158]. Depending on the chemical species of conotoxins, they function as inhibitors of voltage-gated calcium channels (e.g., ω-conotoxins), Nav, nicotinic acetylcholine receptors, serotonin receptors (e.g., σ-conotoxins), NMDA receptors (e.g., conantokins), G-protein-coupled receptors (e.g., ρ-conopeptides), and neurotransmitter transporters (e.g., χ-conopeptides), and so on [159]. For example, μ-conotoxins elicit a sodium channel inhibition through the direct pore block overlapping with tetrodotoxin at site 1, whereas ι-conotoxins enhance the channel opening by shifting the voltage dependence of the sodium channel activation to more hyperpolarized potentials. While α-conotoxins are selective antagonists of the nicotinic acetylcholine receptors, the conopeptide ρ-TIA with 19 amino acid residues inhibits α1-adrenoceptors. It is interesting that unpaired cysteine residues in conotoxins undergo posttranslational modifications, such as ADP-ribosylation [160], lipidations [161], nitrosylation [162], or cysteinylation [163]. These modifications may be involved in additional functionality, stabilization, subcellular localization, and detoxication.

μ-Conotoxins are peptides that are composed of 16~26 amino acid residues, structured by three disulfide bridges. They belong to the M superfamily of conopeptides (six cysteine residues, organized as CC–C–C–CC). The μ-Conotoxin bind to the extracellular S5–S6 loop of Nav, like tetrodotoxin and saxitoxin, although they discriminate further between Nav subtypes, having a higher affinity to the mammalian brain subtype Nav1.2 and the skeletal subtype Nav1.4 than to Nav1.7 and Nav1.8. This difference indicates that the binding sites of tetrodotoxin and μ-conotoxins only partially overlap and involve multiple Nav residues in the case of the larger μ-conotoxins, whereas the tetrodotoxin binding is crucially defined by relatively few residues in the pore of the Nav. The μO-conotoxins possess three disulfide bridges and belong to the O superfamily (six cysteine residues, organized as C–C–CC–C–C). The μO-conotoxins act as inhibitors of sodium channel conductance. Although the binding site of μO-conotoxins remains yet to be fully defined, it overlaps at least partially with those of the δ-conotoxin at the DIV of Nav (binding site 6). Another site is the voltage sensor of DII, which is shared with scorpion β-toxin (binding site 4). It is suggested, therefore, that the interaction of different toxins with a single region of the channel could be responsible for the opposite effects on the conductance. μO-conotoxins function as inhibitors of Nav, while δ-conotoxins and scorpion β-toxins function as activators of Nav [159]. Considering these facts, the Conus species protect themselves from the attack of the conotoxins through the mutation of the target sites and the sequestration and/or post-translational modification of the toxins [157,164]. 

The phylum of Cnidaria is the oldest animal venomous lineage. Its venom is a complex mixture of toxic compounds, including enzymes, pore-forming toxins, and neurotoxins. Actinoporins are the most abundant cnidarian pore-forming toxins, with a molecular weight of about 20 kDa, lacking in an intramolecular disulfide bridge. They specifically bind to sphingomyelin in the lipid membrane, and form oligomeric transmembrane pore, causing an osmotic imbalance and cell death. Most sea anemone species produce different isoforms of a specific actinoporins, which differ in isoelectric point, molecular weight, and cytolytic activity [165,166,167]. Actinoporins are biosynthesized as their prepropeptides, comprising of about 34 amino acid residues, which include the signal peptides of 19~21 amino acid residues. Intriguingly enough, although the actinoporins specifically target the sphingomyelin in the cell membrane, this lipid in sea anemones is replaced by its phosphono analogue. That is, the sphingomyelin possesses a phosphonocholine head group, to which the actinoporins cannot bind, and consequently, makes the sea anemones resistant to their own toxin [168].

Aerolysin-like pore-forming toxins are found mainly in pathogenic bacteria, but also in sea anemones and hydra. Hydralysins, pore-forming proteinous toxins from hydra, show paralytic, cytolytic, and hemolytic activities. They are secreted into the gastrovascular cavity immediately after the engulfment of prey, where they are bound to membranes of the ingested prey. However, the hydra itself is protected from the effect of its own lytic toxins, because hydralysins do not bind to hydra membranes, probably because of the lack of the receptor [169]. 

## 4. Toxins from Terrestrial Animals

Snake venoms are complex mixtures of organic and inorganic compounds that act on a variety of specific metabolic and physiological targets of prey, victims, and predators, assisting in feeding and defense [170,171]. The organic compounds are proteins/peptides in nature, including acetylcholine esterases, complements, disintegrins, defensins, growth factors, nucleases, nucleotidases, metalloproteinases, phospholipase A_2_, proteinase inhibitors, and others [172,173].

**Figure 2:**
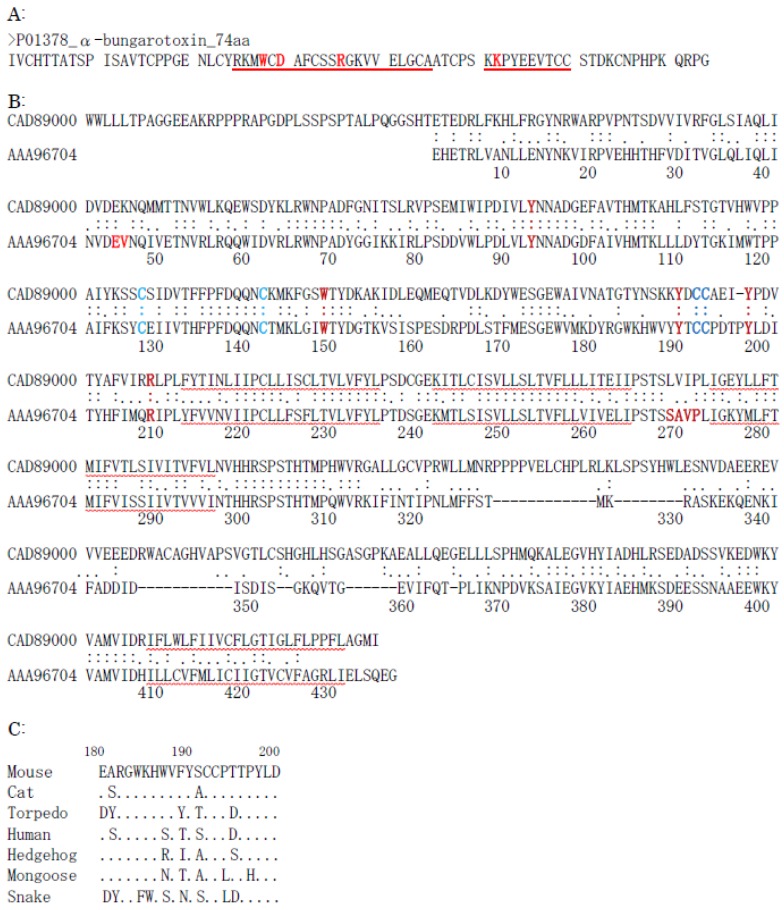
(**A**) Amino acid sequence of α-bungarotoxin (GenBank accession No. P01378). Amino acids involved in the binding to thr acetylcholine receptor are marked with bold red. Red underlines indicate loop II and loop III, respectively. (**B**) Comparison of amino acid sequences of human α2-acetylcholine receptor (GenBank accession No. CAD89000) and *Torpedo marmorata* α-acetylcholine receptor (GenBank accession No. AAA96704). Amino acid residues involved in the ligand-binding are marked with bold red. Wavy red underlines show the trans-membrane regions. (**C**). Comparison of critical amino acid residues of acetylcholine receptors from species that are sensitive or resistant to α-bungarotoxin. Dots indicate the same amino acid residues as that of a mouse.

α-Bungarotoxin is an α-neurotoxin consisting of 74 amino acid residues with five disulfide bridges (GB No. P60615). It is isolated from the snake venom of *Bungarus multicinctus* and binds to the postsynaptic nicotinic acetylcholine receptor at the neuromuscular junction, almost irreversibly. The specific high-affinity binding of α-bungarotoxin to the acetylcholine receptor requiresfive amino acid residues of the C-terminal, and several amino acids that are located near the end of loop II, such as Trp28, Asp30, Arg36, and Lys52 (Figure 2A) [174,175].

Acetylcholine receptors are divided into two types, nicotinic and muscarinic receptors. Nicotinic acetylcholine receptors are pentameric structures consisting of five subunits that are arranged to create a cylindrical complex, forming an ion channel [176]. There are 12 neuronal specific subunits, that is, α2 to α10, and β2 to β4. Depending on the combination of subunits, the structural and functional diversities arise. All of the subunits have a conserved extracellular large N-terminal domain of about 200 amino acids, distinct and conserved; three transmembrane domains; a cytoplasmic loop of various size and amino acid sequence; and a fourth transmembrane domain with a variable extracellular C-terminal sequence. Snake toxins, such as α-cobra toxin and α-bungarotoxin, are bound only to the α-type subunit of acetylcholine receptor. These toxins bind to a hydrophobic pocket that is formed at the interface between the α-subunit and the adjacent subunit. For the ligand-binding, the disulfide bridge (Cys-loop) that is formed between Cys128 and Cys142, and the Cys–Cys pair at 192 and 193, are required. In addition, hydrophobic aromatic amino acids, including Tyr93, Trp149, Tyr190, and Tyr198, are involved in ligand binding. Glu45 and Arg209 are present in every member of the Cys-loop receptor family and they form a common link between the ligand-binding site and channel, including Val46, Ser269, and Pro272 (Figure 2B) [177,178,179].

All of the acetylcholine receptors from species that are sensitive to α-bungarotoxin, such as mice, have a tryptophan at position 187 and an aromatic amino acid residue at position 189. In those from the species that are less sensitive, such as snakes, mongooses, hedgehogs, and humans, these two amino acids are replaced by non-aromatic residues (Figure 2C). In addition, the acetylcholine receptor of the venom-resistant mongooses that feed on snakes has several other mutations, such as Ser191 to Ala, Pro194 to Leu, and Pro197, to His (Figure 2C) [180]. Furthermore, the Asn187 in mongooses and Asn189 in snakes are glycosylated [181]. However, although these mutated acetylcholine receptors do not bind α-bungarotoxin, they still retain their cholinergic properties. Resistance to α-bungarotoxin is believed to have evolved at least four times in mammals, as a consequence of the changes to the nicotinic acetylcholine receptor molecule to which the toxin binds. It is concluded, therefore, that snakes and some mammals, such as honey badgers, hedgehogs, mongooses, pigs, and humans, that are resistant to the attack of snake venom, have mutated and/or modified the acetylcholine receptors as the strategy of the resistance against snake venom [182,183,184]. α-Bungarotoxin acts also as an antagonist to the GABA_A_ receptor [185].

Bothropstoxin-II comprising of 138 amino acid residues (GB No. P45881) is a phospholipase A_2_ that is isolated from *Bothrops jararacussu* snake venom, which induces platelet aggregation and ATP release reaction. The induction is shown to be evoked through multiple signal transduction pathways using several specific inhibitors, including genistein and staurosporine [186]. 

Atrolysin A is a zinc metalloproteinase that is isolated from the venom of the western diamondback rattlesnake, *Crotalus atrox*. It consists of 419 amino acid residues (GB No. Q92043) and shows proteolytic and hemorrhagic activities. Interestingly, both the proteolytic and hemorrhagic activities are partially inhibited by the opossum serum oprin, and completely inhibited by the opossum serum [187]. However, both the proteolytic and hemorrhagic activities of atrolysin B are completely inhibited by oprin. Oprin is homologous to human α-1B-glycoprotein. Atrolysin B is a zinc metalloproteinase that is isolated from the venom of the western diamondback rattlesnake, *Crotalus atrox*. It consists of 414 amino acid residues (GB No. Q90391). The inhibitory activity of the opossum serum may be related to the protection against snake venom [188,189]. In this connection, it is interesting that some opossums belonging to the family *Didelphidae* can eat pitvipers with impunity [190]. Botrocetin, one of the components in snake venom, is a non-enzymatic protein that causes the von Willebrand factor-dependent aggregation of platelets. Intriguingly, some amino acid residues within the botrocetin-binding regions in the von Willebrand factors are substituted in opossums. The prevention of the binding of botrocetin to the von Willebrand factor in opossums may be one of the reasons for the resistance. However, as snake venom contain dozens of toxic compounds, the evolution of the resistance requires adaptive changes at multiple loci.

Micrurotoxin 1 and micrurotoxin 2 are two toxins that are present in the Costa Rican coral snake’s venom that bind tightly to GABA_A_ receptors [191]. Both of them consist of 64 amino acid residues, with five disulfide bridges (GB Nos. C0HJR1 and C0HJR2). The GABA_A_ receptors belong to the pentameric Cys-loop superfamily of ligand-gated ion channel receptors, which encompasses the nicotinic acetylcholine, glycine, and serotonin receptors [178]. The mutation of His33 in micrurotoxin 2 to serine, impairs its function, indicating that this locus is vital for toxin activity. On the other hand, the micrurotoxin 1 function is influenced by mutations in the loop-C [192,193,194] of the α1 subunit of the GABA_A_ receptor [191], indicating that loop-C is involved in the interaction between micrurotoxin and the GABA_A_ receptor, as in the cases of toxins and nicotinic acetylcholine receptors.

Scorpion venom are highly complex mixtures of small peptides, proteins, mucoproteins, amino acids, biogenic amines, lipids, carbohydrates, and inorganic salts. Among them, non-disulfide-bridged peptides are attractive compounds, because they show antimicrobial, antimalarial, immunosuppressing, and anticancer activities, and may be relevant for the development of pharmaceutical drugs [195]. Another toxin is neurotoxins, and they are disulfide-bridged peptides with a significantly constrained structure. They act on various ion channels in excitable membranes, including sodium channels, potassium channels, calcium channels, and chloride channels [196,197]. This process is thought to have developed in response to the extended positive selection via predator-prey interactions.

Scorpion neurotoxins affecting Nav are functionally divided into α- and β-toxins, according to their primary actions on these channels. α-Toxins target the Nav receptor site 3, inhibiting channel inactivation, while β-toxins bind to site 4 of the Nav receptors [71,137,138,198]. Both toxins contain 60~80 amino acids that are linked by four disulfide bridges.

LqhII and LqhIII are α-toxins that are isolated from the venom of *Leiurus quinquestriatus hebraeus,* consisting of 64 amino acids with 4 disulfide bonds and 67 amino acids with 4 disulfide bonds, respectively (GB Nos. P59355 and P56678, respectively). The LqhII sequence reveals only one of each substitution of N-terminal and C-terminal amino acid, as compared to AaHII (GB No. P01484), which is isolated from *Androctonus australis*. LqhII and sea anemone toxins are shown to bind to the overlapping region comprising receptor site 3 on the rat brain and insect sodium channels (DIV S3-S4). The mutation of some amino acids in this region makes Nav resistant to scorpion α-toxin LqTx [199,200]. Interestingly, LqhII shows toxicity to mice, comparable to that of AaHII, while LqhII shows a 3.2-fold higher toxicity to cockroaches, as compared to AaHII, indicating that the N-terminal and C-terminal amino acids determine the species specificity of toxicity of the two toxins. LqhIII has an 80% sequence identity with the α-like toxin BomIII (GB No. P13488). LqhIII shows about a 2-fold lower toxicity to mice than BomIII, but is about 2-fold more toxic to cockroaches than BomIII. Thus, relatively minor changes in the sequence of scorpion toxins affect their relative species selectivity [201]. Moreover, it is shown that the tolerance of insects to a scorpion toxin AaIT occurs at both the pharmacokinetic and pharmacodynamics levels [202]. The CssIV from *Centruroides suffuses* belongs to the class of scorpion β-toxins (GB No. P60266), and shifts the voltage-dependent activation to more negative membrane potentials, leading to repetitive firing in muscles and nerves. This activity depends on the binding to DI S5-S6, DII S1-S2 and DII S3-S4, and DIII S5-S6. The mutations of the ritical amino acids in these regions result in a reduction of voltage-sensor trapping activity [203]. 

As for the resistance to scorpion toxins, several papers were reported. Rowe et al. reported that bark scorpion toxin induces pain in many mammals, including house mice and humans, by activating Nav1.7, but it has no effect on Nav1.8 [204]. On the other hand, for grasshopper mice, *Onychomys torridus,* Nav1.8 has several amino acid mutations, which bind bark scorpion toxins and inhibit Na^+^ currents, inducing analgesia. Especially, the mutations of amino acid residues in the DII SS2-S5 linker region of Nav1.8 are involved in this phenomena. Thus, by using a toxin that is bound to a non-target Nav, the resistance in grasshopper mice is aided by enhancing the interaction between toxin and receptor, such that the physiological consequences of the toxin binding are altered to the benefit of the targeted animal [204]. The long-eared bat (*Otonycteris hemprichii*) and pallid bat (*Antrozous pallidus*) can eat scorpions without harmful effects, although the exact resistance mechanisms to the toxins have not been clarified [205,206]. Legros et al. reported that the venom from the scorpion, *Androctonus australis*, is pharmacologically inactive on K^+^ channels and on the Nav from this scorpion [207].

**Figure 3:**
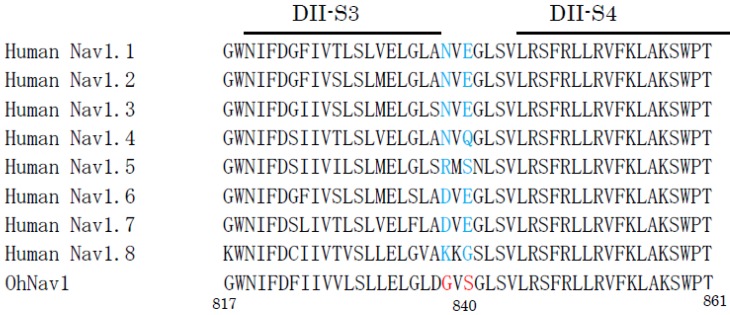
Comparison of amino acid sequences in site 3 of the human sodium channels and that of *Haplopelma schmidti* (OhNav1). The critical residues are marked with blue and red. The amino acid numbering is according to OhNav1.

Spider venom is made up of complex mixtures of polyamines; lectins; defensins; enzymes, such as proteinases; phospholipases and hyaluronidases; neurotoxins; and others. They act as receptor and/or ion channel toxins [208,209,210,211,212], antibacterial substances [213], and potentiators of erectile function [214], and so on. On the level of sequence identity and inter-cysteine spacing, spider toxins that target Nav channels are divided into 12 families [211]. Huwentoxin-IV (GB No. AAP33074) is a sodium channel inhibitor that is isolated from the venom of the Chinese tarantula, *Ornithoctonus huwena*, and is composed of 35 amino acid residues with three disulfide bridges. It belongs to the family 1. It preferentially inhibits the neuronal subtype Nav1.7 and is docked at the receptor site 4, which is located at the extra-surface DII S3-S4 linker region [215]. From the analysis of the mutants of huwentoxin-IV, it is suggested that the polar residues threonine-28, arginine-29, and glutamine-34 in the C-terminal play crucial roles in the interaction of huwentoxin-IV and Nav [216]. On the subesophageal ganglion neurons from the tarantula, at least three types of voltage-gated ion channels are co-expressed, namely, calcium channels, two types of potassium channels, and tetrodotoxin-sensitive sodium channels. Interestingly, these ion-channels are relatively insensitive to their own toxins. As for the sodium channels, huwentoxin-IV preferentially inhibits Nav1.7. However, the affinity of huwentoxin-IV for the tarantula tetrodotoxin-sensitive sodium channel is over 120-fold lower than for the human Nav1.7. A comparison of the amino acid sequences in the site 4 regions of the Nav1.7 of human and tarantula reveals that two crucial residues (Asp837 and Glu839) are substituted by two neutral residues (Gly837 and Ser839; GB No. ABH12275; Figure 3). This indicates that the substitutions of the acidic amino acids in the critical region with neutral amino acids may cause the self-resistance to their own toxin [216,217]. Jingzhaotoxin-I is a 33 amino acid residue inhibitor cysteine knot motif peptide that has been separated from tarantula, *Chilobrachys jingzhao*, venom. It inhibits the tetrodotoxin-resistant Nav that is expressed in mammalian cardiac myocytes and tetrodotoxin-sensitive Nav in mammalian sensory neurons, but does not inhibit the tetrodotoxin-resistant Nav in mammalian sensory neurons [218]. The toxin interacts with site 3, located at the extracellular S3–S4 linker of DIV. Interestingly, the single mutation of two amino acid residues (Asp1609 and Lys1613) in the S3–S4 loop of the DIV decreases the sensitivity of the toxin for the human Nav channel [219]. However, it is not clear how these mutations are related to the resistance to the toxin in the producing tarantula.

Amphibians like poisonous frogs are protected by an exocrine defense system that is composed of cutaneous poison glands, which are specialized cells secreting a variety of defense chemicals [220]. The defensive chemicals include alkaloids, biogenic amines, bufadienolides, and so on [221]. The poisonous frogs, such as Bufoniae and Dendrobatidae, sequester the lipophilic alkaloids, such as indolizidines, from dietary sources like mites and ants [222,223,224]. However, the resistance mechanism in poisonous frogs is only poorly understood. The resistance to batrachotoxin is a modification of the target, voltage-gated sodium channels [225,226]. Table 2 shows the toxin resistance-related genes in marine and terrestrial animals. The self-defense mechanisms in marine and terrestrial animals are mostly due to the modification or mutation of the targets. However, the toxins that have been discussed here are mainly peptides or proteins in nature, except for tetrodotoxin, saxitoxin, lipophilic alkaloids, and batrachotoxin. Producer animals also sequester the toxins or the defensive chemicals in specialized cells [221]. This is another important strategy for defense against themselves (self-defense) and intruders, and may be essential for a predator–prey struggle. In saxitoxin, the producer microorganisms use transporters as a defense mechanism, while the exposed animal (*Mya arenaria*) uses the mutation of the target, indicating that the defense mechanisms show inter-species and inter-population variation [72] (Table 1 and Table 2). 

## 5. Plant Toxins

Plants produce a vast variety of secondary metabolites, differing in chemical structures and functions. They act as defense, signaling, and immunological compounds in plants, among many others. Camptothecin (CPT) is a water-insoluble tryptophan-derived quinolone alkaloid and is a lead compound for two FDA (Food and Drug Administration) approved antineoplastic drugs, irinotecan and topotecan [227,228]. It was originally isolated from *Camptotheca acuminata* more than 50 years ago [229]. However, it is now known that it is found in at least 16 different plant species, belonging to 13 unrelated genera. Moreover, it is produced not only by endophytic fungi, including *Entrophosphora infrequens*, *Neurospora* sp., and *Fusarium solani* [230,231], but also by endophytic bacteria from *Pyrenacantha volubilis* (*Icacinaceae*) [232]. The endophytic bacteria belong to the *Bacillus* species, and a 5 kb plasmid was isolated from one of the bacteria. It is speculated that the biosynthetic genes are present on the 5 kb plasmid, because the elimination of the plasmid by the treatment with acriflavine results in the loss of the production of CPT [232]. The reason for this remains to be clarified, because the plasmid is too small to cover the biosynthetic gene cluster for CPT.

**Figure 4:**
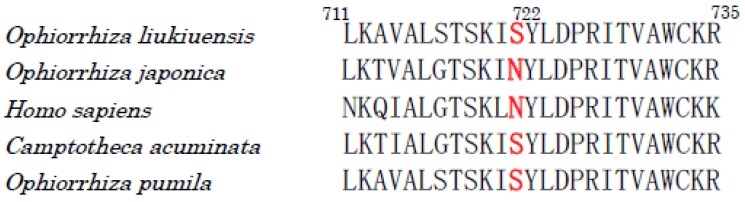
Comparison of the amino acid sequences of CPT is of the CPT-producing and CPT-nonproducing plants and humans. *Ophiorrhiza liukiuensis*, *Camptotheca acuminate*, and *Ophiorrhiza pumila* are CPT-producing plants, while *Ophiorrhiza japonica* and *Homo sapiens* are nonproducing. GenBank accession numbers are BAG31374, BAG31376, BAG31373, BAG31375, and AAA61206, respectively. The critical amino acid residues for the resistance (aspragine and serine) were marked with red.

CPT is an inhibitor of eukaryotic topoisomerase I. It is also toxic to most higher-plants. So, it is necessary for the producing plants to protect themselves from the attack of their own toxin. A comparison of the amino acid sequences of topoisomerase Is of the CPT-producing and CPT-nonproducing plants revealed that asparagine at 722 (numbered according to human topoisomerase I) in the nonproducing plants, such as *Ophiorrhiza japonica*, and humans is substituted by serine in the CPT-producing plants, such as *Ophiorrhiza pumila*, *O. liukiuensis*, and *Camptotheca acuminata* (Figure 4), suggesting that the mutation of Asn722Ser is responsible for the self-resistance to CPT in the producing plants [233,234].

Sclareol is a labdane-type dipertene that is detected on the leaf surface of the *Nicotiana* species. It shows antifungal activity and also inhibits plant growth. It is reported that the drug resistance-type ATP-binding cassette (ABC) transporter, NpPDR1, is involved in extracellular sclareol secretion in *Nicotiana plumbaginifolia*, and the expression of NpPDR1 makes it resistant to sclareol. Interestingly, NpPDR1 is constitutively expressed in the trichome, where the biosynthesis of sclareol occurs essentially [235,236,237]. After biosynthesis, phytotoxins are often stored in specialized organs [238].

ABC transporters are involved in the transportation of defense molecules, sequestration of xenobiotics and intracellular metabolites to the vacuole, and many others [239,240,241]. Berberine is a benzylisoquinoline alkaloid and is used as an antidiarrhetic and antimalarial drug. *Coptis japonica* accumulates berberine exclusively in the vacuoles (sequestration), whereas *Thalictrum minus* secretes biosynthesized berberine outside of the cells (excretion). The ABC transporter CjABCB1/CjMDR1 and a P-glycoprotein-like ABC transporter are involved in these processes [242,243]. However, a proton-antiporter may also mediate the membrane transport of berberine [244]. Vinblastine and vincristine are indole alkaloids that are isolated from *Catharanthus roseus* and are used as anticancer drugs that disrupt the microtubule formation, and interfere with amino acid and glutathione metabolisms, and nucleic acid and lipid biosynthesis [245,246]. In *C*. *roseus*, an ABC transporter CrTPT2 functions as an efflux transporter of catharanthine to the cell surface. Catharanthine is a biosynthetic precursor of vinblastine and vincristine [247,248]. Flavonoids are a major class of plant secondary metabolites. Some flavonoids show anti-oxidation, anti-inflammatory, and antitumor activities [249]. To avoid their toxic effects, producing plants sequester flavonoids within vacuoles by transporting them with the multidrug resistance-related protein subfamily of ABC transporters [250].

Sanguinarine is a benzophenanthridine alkaloid with cytotoxic properties, such as the induction of oxidative DNA damage and rapid apoptosis [251]. It also inhibits the growth of cultured cells of *Nicotiana* and *Arabidopsis*. So it is either accumulated in the vacuole or excreted into the cell wall for self-protection [252,253]. In addition, it is reduced to the less toxic dihydrosanguinarine [254,255,256]. Coniine is a piperidine alkaloid isolated from *Conium maculatum* and is known as the killer of Socrates in 399 BC [257]. It is a nicotinic acetylcholine receptor antagonist, which leads to the inhibition of the nervous system and the antinociceptive effect [258]. Interestingly, the cabbage looper (*Trichoplusia ni*) larvae that are raised on coniine and γ-coniceine-enriched diets do not show any effect on the growth and only a slight reduction of development time. The three reasons are proposed to explain these responses, namely: (1) a decreased consumption rate, (2) efficient excretion of ingested alkaloids unmetabolized in frass, and (3) partial detoxification of alkaloids by cytochrome P450 [259]. This proposal is supported by the fact that the larval growth is decreased in the presence of piperonyl butoxide, a cytochrome P450 inhibitor.

The pyrrolizidine alkaloids represent a class of plant secondary metabolites [260,261,262], and are strongly hepatotoxic, pneumotoxic, and teratogenic to most vertebrates and invertebrates. They are most likely produced as a chemical defense mechanism against herbivores. Some pyrrolizidine alkaloids, such as clazamycins and jenamidines, are biosynthesized by Gram-positive and Gram-negative bacteria [263]. *Senecio* species biosynthesize pyrrolizidine alkaloids in unique patterns, as senecionine N-oxide, as a common intermediate in the roots. Then, species-specific alkaloids are thought to be produced in shoots. These alkaloids are stored in vacuoles in the form of mainly their N-oxides [264]. On the other hand, a protective role of plant pyrrolizidine alkaloids is observed in specialized herbivorous insects. A number of insect herbivores have evolved adaptations not only to overcome the defense barrier of pyrrolizidine alkaloid-protected plants, but also to sequester and utilize the alkaloids for their own defense against predators. For example, the larvae of the European cinnabar moth, *Tyria jacobaeae*, sequester the alkaloids from their larval host plant *Senecio jacobaea*. Larvae raised on a pyrrolizidine alkaloid-free diet prove consistently palatable to wolf spiders, whereas the larvae and adults containing the alkaloids were rejected [265,266]. Leaf beetles of the genus *Oreina* are another example of pyrrolizidine alkaloid sequestration for insect defense. Leaf beetles release their defense compounds from special exocrine glands that are located in the elytra and pronotum. Most insects with the alkaloid-sequestering species store and maintain the alkaloids as N-oxide. The N-oxides are less toxic than the parent alkaloids, so it is more convenient for self-protection. In arctiids, the sequestered pyrrolizidine alkaloid N-oxide are found in all of the tissues, but preferentially in the integument [267,268]. 

Steroidal glycoalkaloids, such as α-solanine and α-chaconine, found in solanaceous food plants like potato and tomato, are antinutritional factors for humans. They cause gastrointestinal and neurological disorders and are lethal for humans at high concentrations. They disrupt membranes and inhibit acetylcholinesterase activity [269]. The steroidal glycoalkaloids consist of two structural components, the aglycone, a cholesterol-derived compound; and a carbohydrate side-chain. The biosynthetic gene clusters of α-tomatine and α-solanine were cloned [270,271]. The endogenous enzymes with glycosidase activity remove sugar molecules from the saccharide moiety of α-tomatine, creating a less cytotoxic compound, α-tomatidine. This detoxification mechanism was also observed in *Fusarium oxysporum* and *Cladosporium fulvum* [272,273], indicating that the presence of glycosidases in the plants and fungi are associated with modulation of the toxicity of steroidal alkaloids in the defense response. This indication is supported by the fact that the aglycones solanidine and tomatidine produce only a slight to negligible inhibition of acetylcholinesterase activity [269,274], and that the glycosylation by GAME1, a gene that is involved in the biosynthesis, is crucial to prevent the toxic effect of the alkaloids to the plant cells [275]. On the other hand, benzoxazinoids are stored as biologically inactive glycosides that are cleaved by β-glucosidase upon attack, releasing the active aglycones [276]. The aglycones are active against bacteria, fungi, and herbivores. The iridoid glucosides, such as aucubin and catalpol, are other examples. After the attack of herbivores, iridoid glucosides are cleaved by glucosidases to the toxic terpenoid aglycones [277,278].

More than 3000 plants species, such as almond and sweet cherry, use hydrogen cyanide (HCN) as a fast-acting, powerful toxin to protect their seeds and leaves against attack from herbivores. The cyanogenic glucosides, prunasin and amygdalin, release HCN upon cell wounding. Tissue disruption brings together both cyanogenic glycosides and the HCN-releasing enzymes, β-glucosidase and hydroxynitrile lyase, which are stored in separate compartments in the intact plant cells [279,280]. Glucosinolates are produced by Brassicaceae, such as cabbage, rapeseed, and radish. Upon tissue disruption, glucosinolates are cleaved by myrosinase, a glucosidase, to form toxic isothiocyanates [281]. These binary glycoside and glycosidase systems are referred to as two-component plant chemical defense [278].

**Figure 5:**
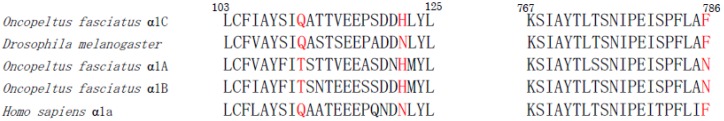
Comparison of amino acid residues of three isoforms of *Oncopeltus fasciatus* Na^+^/K^+^-ATPases, and those of *Drosophila melanogaster* and *Homo sapiens*. The critical residues are marked with red. GenBank accession numbers are AFU25689, AFU25688, AFU25687, AAC05260, and NP_000692, respectively. The critical amino acid residues for the binding of ouabain were marked with red.

Cardenolides are composed of aglycones of steroid structures that are derived from terpenoids and sugars [282]. Although mostly recognized as plant compounds, cardenolides are produced via the cholesterol pathway in animal tissues. Ouabain and digoxin are the typical endogenous cardiac glycosides. They are strong inhibitors of the Na^+^/K^+^-ATPases. Ouabain is toxic to locusts or cockroaches, while to caterpillars of the tobacco hornworm ouabain is tolerated. This insensitivity is explained by the high concentration of K^+^ in the lepidopteran hemolymph. K^+^ has an antagonistic effect to the ouabain binding to the Na^+^/K^+^-ATPase. On the other hand, the caterpillars of the monarch butterfly sequester cardenolides from its apocynaceous host plants [283]. In addition, mutations of critical amino acid residues of the target (Na^+^/K^+^-ATPase) lower the sensitivity of the monarch butterfly (*Danaus plexippus*) and the milkweed bug (*Oncopeltus fasciatus*) to cardenolides. For example, the milkweed bug has three copies of the Na^+^/K^+^-ATPase α1 subunit gene, α1A, α1B, and α1C. The α1C knockdowns with RNAi cause difficulties in motor function and have a reduced survival rate, indicating that the α1C gene is the most important for survival. On the other hand, although the α1A or α1B knockdowns with RNAi sustain the normal survival rate, they are no longer able to tolerate cardenolides. The comparison of amino acid residues that are involved in the binding of ouabain [284] indicates that substitutions of Gln111Thr, Asn122His, and Phe786Asn mediate insensitivity to cardenolides (Figure 5) [285,286,287]. Furthermore, it is reported that the oleander hawk moth (*Daphnis nerii*) uses the perineurium as a diffusion barrier (restriction) for polar cardenolides like ouabain and efflux transporters (exclusion), for non-polar cardenolides like digoxin. As quinidine and verapamil inhibit the barrier, P-glycoproteins-like transporters are suggested to be involved in the barrier. These results suggest that the lepidopteran perineurium functions as a diffusion barrier for polar cardenolides and forms an active barrier for non-polar cardenolides [288]. 

Nicotine, an alkaloid derived from the leaves of tobacco plants (*Nicotiana tabacum*, *Nicotiana attenuate,* and other *Nicotiana* species), is the primary addictive agent in tobacco products and binds to nicotinic acetylcholine receptors [289]. Nicotine is stored in the trichomes of tobacco leaves (sequestration). *N. attenuate*, a species of wild tobacco, is attacked by larvae of both specialist (*Manduca sexta*) and generalist (*Spodoptera exigua*) lepidopteran herbivores. *M. sexta* is highly tolerant to nicotine. Interestingly, ingestion of nicotine and its N-oxides to *M. sexta* larvae induces cytochrome P-450 CYP6B46. Nicotine-induced CYP6B46 is used to efflux midgut-nicotine into the hemolymph and it facilitates nicotine exhalation. Nicotine, but not nicotine-N-oxide, deters predatory wolf spiders. On the other hand, the *S*. *exigua* larvae oxidizes nicotine and are more susceptible to predation by wolf spiders [290].

The defenses of plants to insects and pathogens are initiated by the recognition of insect oral secretion and signals from injured plant cells. These early events include damage-induced ion imbalance, variations in membrane potentials, Ca^+^-signaling, production of reactive oxygen species, kinase activities, and phytohormones [291,292,293,294,295]. *N*. *attenuate* α-DIOXYGENASE1 is an oxylipin-forming gene that is elicited during herbivory by fatty acid-amino acid conjugates, which are contained in oral secretion of *M. sexta*. *N*. *attenuate* specifically accumulates 2-hydroxylinolenic acid during feeding by *M. sexta* larvae. α-DIOXYGENASE1-silenced plants are less resistant to a *M. sexta* attack, indicating that 2-hydroxylinolenic acid, produced from linolenic acid by attack-activated-α-DIOXYGENASE1, participates in defense activation during insect feeding [296].

Menthol is a cyclic monoterpene alcohol, which possesses cooling characteristics and is a major constituent in the essential oil of *Mentha canadensis* L. It acts upon the transient receptor potential melastatin family member 8 (TRPM8) receptors by rapidly increasing the intracellular calcium and mobilizing the calcium flux. Aside from its cold-inducing sensation capabilities, menthol exhibits cytotoxic effects in cancer cells, induces reduction in malignant cell growth, and engages in synergistic excitation of GABA receptors and sodium ion channels, resulting in analgesia. It is often stored in trichomes from which it is released upon cell rupture [297,298].

Lupins produce quinolizidine alkaloids, such as albine, lupanine, and multiflorine, in leaf chloroplasts, export them via the phloem all over the plant, and the accumulate in epidermal tissues, especially in reproductive organs. Quinolizidine alkaloids are known to interfere with the nervous systems of animals. As for lupins, alkaloid-rich and alkaloid-free varieties (sweet lupins) are known [299,300]. It is shown that aphid generalists such as *Myzus persicae* only suck on sweet lupins, and not on alkaloid-rich varieties with high alkaloid contents in the phloem. On the other hand, specialist aphids, such as *Macrosiphum albifrons,* live on lupins, sequester the dietary alkaloids, and use them as a defense against predators [301]. Many other animals show a similar discrimination. Table 3 shows the toxin resistance-related genes in plant. In plants, sequestration in specialized organs like vacuoles and the related transporters are the major mechanisms of the resistance. These mechanisms are used as the defense, not only in producer plants, but also in herbivore animals such as insects. In addition, the mutation of targets such as topoisomerases and chemical conversion of glycoside to aglycones or reverse are also observed. 

## 6. Toxins from Fungi

Fungi, in particular the fruiting bodies of higher fungi, are potential victims of attack by fungivores and microorganisms. The fruiting bodies of mushrooms are a rich source of secondary metabolites with unusual chemical structures. Aflatoxins are among such metabolites. They are produced mainly by *Aspergillus flavus* and *A. parasiticus*. The four major natural aflatoxins are known as aflatoxins B_1_, B_2_, G1, and G_2_. Aflatoxin B_1_ and aflatoxin B_2_ are hydroxylated and excreted in the milk as less toxic aflatoxins, M_1_ and M_2_. Aflatoxin B_1_ is metabolized and activated in the intestine and liver by cytochrome P450 to aflatoxin B_1_-8,9-epoxide. Aflatoxin epoxide is highly electrophilic and reacts with the DNA guanine moiety to form covalent bonds at the N-7 guanine residue, leading to depurination and carcinogenesis. Aflatoxin epoxide also attacks mitochondrial DNA and disrupts ATP production. These damages lead to hepatic fibrosis, decreased liver function, and cancer. Differences of sensitivity to toxicity of aflatoxin B_1_ are due to the differences in its metabolism [302,303]. The aflatoxin biosynthetic gene clusters ranging 82 kb were cloned from *Aspergillus flavus* [304,305,306] and *A. parasiticus* [307]. Although *aflT* codes a membrane-bound protein with homology to antibiotic efflux pumps and is presumed to be involved in aflatoxin secretion, the disruption of this gene does not affect theaflatoxin formation [306,307]. Other genes that are related to self-resistance are not detected, although numerous genes for ABC and MFS transporters are present in the genomes [304,308]. Sterigmatocystin, a mycotoxin that is produced by the *Aspergillus* fungi, and causing a carcinogenic, mutagenic, and teratogenic effect as aflatoxins, is biosynthesized as a precursor of aflatoxin A [304,309,310].

Ochratoxins and citrinin are produced by several species of the genera *Aspergillus* and *Penicillium*. The fungi producing ochratoxins and citrinin are commonly encountered in animal feed and human food. Ochratoxins are pentaketide-derived dihydroisocoumarin moieties that are peptide-bonded to phenylalanine derivatives. Three ochratoxins are known and the order of toxicity is ochratoxin A, ochratoxin B, and ochratoxin C. The most sensitive effects of ochratoxin A are on the kidney, causing nephropathy and urinary tumors. The ochratoxin biosynthetic gene clusters were cloned from five species, namely, *Aspergillus steynii*, *A. westerdijkiae*, *A. niger*, *A. carbonarius*, and *Penicillium nordicum* [311,312,313,314,315,316]. A comparison of the five clusters revealed that the central part of the clusters consists of five ORFs, namely, halogenase, bZIP transcription factor, cytochrome P450, non-ribosomal peptide synthetase (NRPS), and polyketide synthase, although the genes in the flanking regions are different [314]. Interestingly, the gene cluster from *P. nordicum* contains ORFs that are homologous to an organic anion transporter and a nitrate transporter [312,313]. The former transporter was reported to be responsible for the transport of ochratoxin into the outside of the cell. Citrinin is a mycotoxin that is produced by genera *Penicillium*, *Aspergillus*, and *Monascus*, and shows a nephrotoxic activity. The biosynthetic gene cluster was cloned as a 43kb DNA fragment from *M. aurantiacus* and *M. purpureus* [317,318]. The Orf5 codes for a putative membrane transport protein.

Fusarium head blight is a serious fungal disease of grains that is caused by the infection of a range of *Fusarium* fungi. *Fusarium*-infected grains are often contaminated with mycotoxins such as trichothecenes, like nivalenol and deoxynivalenol (vomitoxin); fumonisins; and zearalenones. These contaminated toxins are also hazardous to humans and livestock. *Fusarium* species produce both sexual and asexual spores. These spores are resistant to environmental stresses, and play important roles in the development and propagation of the *Fusarium* species. The determination of the whole genome sequences of *F. graminearum* [319] and various omics analyses [320] clarify the penetration and invasion strategies of *F. graminearum*, and hence, the defensive strategies in the host plants, such as wheat and barley, at the genetic level. As a result, signaling molecules such as salicylic acid, jasmonic acid, and ethylene are elucidated to initiate the signal transduction systems in the defense and pathogenesis systems [321,322]. Furthermore, plant proteins, such as ABC transporters, uridine diphosphate-glucosyltransferases, cytochrome P450s, and glutathione-S-transferases, are shown to be involved in deoxynivalenol detoxication [323]. On the other hand, the trichothecene biosynthetic gene cluster was cloned from *F. graminearum*, where one efflux pump gene (*Tri12*) was identified [324,325], indicating that the efflux pump excretes the trichothecene into the outside of the cells in the trichothecene-producing organisms. *F. graminearum* produces a red pigment, aurofusarin, which is a polyketide derivative. Interestingly, the biosynthetic gene cluster contains an efflux pump gene, although aurofusarin is not a mycotoxin. However, the aurofusarin deficient mutants increase the level of the mycotoxin zearalenone [326]. Zearalenone is a nonsteroidal estrogenic mycotoxin that is produced by several species of *Fusarium* fungi. Zearalenone has major effects on reproduction in females, but it affects the male reproductive system as well [327]. The biosynthetic gene cluster of zearalenone was cloned from *Fusarium graminearum* as a 50 kb DNA fragment [328,329,330]. A gene for monocarboxylate transporter-like protein (GzMCT) is located adjacent to the gene cluster [329], indicating that the transporter may be involved in the excretion of zearalenone from the inside of the cells to the outside. *Clonostachys rosea* is a soil-borne ascomycete and is known to be a potential biological control agent against various plant pathogens, including zearalenone-producing *Fusarium culmorum*. The enzyme zearalenone hydrolase in *C*. *rosea* is shown to be responsible for the transformation of zearalenone to the far less product [331,332]. Fumonisins are a group of mycotoxins that are produced by *Fusarium* species and have been shown to cause liver damage in various species, including primates. The genetic and biochemical analyses of *Fusarium* identified a fumonisin biosynthetic gene cluster, including two transporter proteins [333,334]. The gene clusters were also identified in the *Aspergillus* species, where a gene for the transporter protein was present [335,336].

Penitrem A is an indole diterpene mycotoxin that is produced by the *Penicillium* species. It is biosynthesized through paxilline and secopenitrem. The biosynthetic genes for the penitrems cloned from *Penicillium crustosum* consist of two separate clusters, which contain the transporter gene *ptmT* [337]. The genes *ptmGAQMBCP* are highly homologous to those of the paxilline gene cluster. Roquefortine C is a mycotoxin belonging to a class of naturally occurring diketopiperazines that are produced by the *Penicillium* species. It shows bacteriostatic and neurotoxic activities. Roquefortine C was proposed to be the precursor of meleagrin and neoxaline [338]. The roquefortine/meleagrin biosynthetic gene cluster was cloned from *Penicillium chrysogenum*, where a facilitator superfamily transporter gene was present [339]. Aphidicolin, a fungal diterpene that is isolated from *Cephalosporium aphidicola*, is a specific inhibitor of the DNA polymerase α. The gene cluster for the aphidicolin of the 15.6 kb DNA fragment was cloned and sequenced, which includes the ABC transporter protein composed of 564 amino acid residues [340].

Sirodesmin PL is a phytotoxin that is produced by the fungus, *Leptosphaeris maculans*. It causes a chlorotic lesion on plant leaves, and has antibacterial and antiviral activities. Sirodesmin PL is a member of the epipolythiodioxopiperazine (ETP) class of fungal secondary metabolites. The biosynthetic gene cluster of the 68 kb DNA fragment was cloned and sequenced, containing MDR1 type ABC transporter, SirA. It is supposed to be involved in toxin export and self-protection [341,342,343]. Gliotoxin is produced by several fungi, including *Gliocladium fimbriatum*, *Aspergillus fumigatus*, *Trichoderma*, *Penicillium*, and some *Candida* species. It has an immunosuppressive activity and is a virulent factor of the human fungal pathogens. It is also a member of the epipolythiodioxopiperazine (ETP) mycotoxins. The biosynthetic gene cluster was cloned from *A. fumigatus*, containing a major facilitator superfamily type transporter [342]. In addition, GliT, a gliotoxin reductase, plays an important role in the self-resistance [344,345].

The ergot alkaloids are a family of secondary metabolites that are produced by several orders of fungi in the phylum Ascomycota, and particularly in plant pathogens and plant symbionts of the family, Clavicipitaceae. The alkaloid profiles are also diverse. Their activities are derived from their affinity for receptors for the monoamine neurotransmitters [346]. The activities of ergot alkaloids include vasoconstriction or vasodilation, stimulation of uncontrolled muscle contraction and hallucination, and other effects on the central nervous system. The gene cluster for ergot alkaloids was cloned from *Claviceps purpurea*, extending over 68.5 kb, containing four different nonribosomal peptide synthetase genes [347,348,349]. Now, the biosynthetic gene clusters were cloned in several species of fungi [350,351,352,353,354]. So, 14 genes, that is, *dmaW*, *easF*, *easC*, *easE*, *easD*, *easA*, *easG*, *cloA*, *lpsB*, *lpsA*, *lpsC*, *easH*, *easP*, and *easO*, have been shown to direct steps in the ergot alkaloid biosynthetic pathway of Clavicipitaceae, although only *Periglandula ipomoeae* is known to have all of them. However, no transporter gene was present in these regions [351]. Interestingly, the genes that are common to the clusters encode the enzymes catalyzing early shared biosynthetic steps, whereas those that are unique to the clusters of specific fungi, encode later, lineage-specific steps [347,350,355,356]. It is speculated, therefore, that the responsible genes have been evolved through multiple events of gene duplication, gene gain, and gene loss.

Loline alkaloids are produced by endophytic fungi, protecting host grasses by affecting a large range of insects, providing resistance to vertebrate and invertebrate herbivores, and pathogens and parasites [357]. The two biosynthetic gene clusters (*LOL-1* and *LOL-2*) were detected in the fungal symbiont *Neotyphodium uncinatum*. Nine genes were identified in a 25 kb region of *LOL-1*, and *LOL-2* contained the homologs *lolC-2* through *lolE-2* in the same order and orientation [356,357,358,359,360]. The biosynthetic gene clusters of indole-diterpenes such as paspalinine, paspalitrem A, terpendole C and E, and lolitrem B and E, and of peramine in epichloae and other Clavicipitaceae, were cloned and compared [356,361,362,363,364]. The gene cluster for peramine contains two putative members of the facilitator superfamily of transporter proteins, although it is not clear if these proteins are involved in the peramine excretion. Interestingly, these proteins are conserved in *Fusarium graminearum*, *Neurospora crassa*, *Magnaporthe grisea*, and *Aspergillus nidulans* [362].

Swainsonine is an indolizidine alkaloid that is produced by insect and plant pathogens and symbionts belonging to the order Hypocreales, Chaetothyriales, Onygenales, Pleosporales, and Leotiomycetes. It inhibits, specifically, α-mannnosidase II in the Golgi apparatus, disrupting the endomembrane system of the cells. The biosynthetic gene clusters were cloned from six species, which contained a transmembrane transporter SwnT, except the two endophytes, *Ipomoea carnea* and *Alternaria oxytropis* [365]. Table 4 summarizes the toxin resistance-related genes in the fungi. As for the self-resistance against toxins from the fungi, the predominant strategies are transporters. The transformation of the toxins, like zearalenone, to less toxic derivatives is also reported. However, it seems that much more analyses are needed to clarify the detailed self-resistance strategies in fungi.

## 7. Antibiotic Resistance

Drug resistance, especially antibiotic resistance, is one of the most prevalent and threatening events in public health. The genes of antibiotic resistance are hypothesized to be derived from the antibiotic-producing bacteria, such as *Streptomyces* [366,367,368]. However, thanks to the metagenomic and high-throughput sequencing technologies, the current knowledge on resistome, the collection of the resistance genes [369,370], has expanded tremendously [371,372,373]. The aminoglycoside antibiotics, such as streptomycin, kanamycin, and gentamicin, interfere with protein synthesis by acting on the smaller 30S subunit of the bacterial ribosome, causing bactericidal effects against the pathogens [374]. In addition, the possibility for the treatment of the human immunodeficiency virus infection has been demonstrated [375]. Streptomycin is the first aminoglycoside antibiotic [376]. The aminoglycoside antibiotics are classified into two groups, those whose activities are affected and those not affected by methylation of 16S rRNA [374]. Streptomycin belongs to the latter group. The biosynthetic gene cluster was cloned as a 90kb DNA fragment [377,378] (GB No. AJ862840). It contains two phosphotransferases (StrA and StrK) and the EamA/RhaT family transporter, located adjacent to the cluster. These proteins are supposed to be involved in the self-resistance in *Streptomyces griseus* [379] (GB No. NC_010572).

Kanamycin was isolated from *Streptomyces kanamyceticus* [380] and is the 4,6-disubstituted 2-deoxystreptamine-containing amino glycoside antibiotic, together with gentamicins, tobramycin, and amikacin. Their activities are compromised by methylation of 16S rSNA. The kanamycin biosynthetic gene clusters were cloned [381,382,383,384] (GB Nos. AJ582817, AB164642, and AB254080). They contain genes for aminoglycoside 6′-*N*-acetyltransferase (*kanM*) and 16S rRNA methyltransferase (*kmr*), indicating that they are involved in self-resistance. Furthermore, there are several efflux (KanO and KanN) and ABC transporter proteins (KanS, Kan R and KanQ). Interestingly, kanamycin A and kanamycins B and C are biosynthesized in two different routes [385]. The gentamicin biosynthetic gene clusters were cloned from *Micromonospora echinospora* [381] (GB Nos. AJ575934, AJ628149, and AY524043). Three proteins within the clusters (GtmL/GmrB/GrmO, GtmF/GmrA/GrmA, and GtmK/GenV/GntO) were proposed to be involved in self-resistance. GtmL/GmrB/GrmO and GtmF/GmrA/GrmA are rRNA methyltransferases, and GtmK/GenV/GntO are transmembrane efflux proteins. Tobramycin is 3′-deoxykanamycin B. The gene clusters were cloned from *Streptoalloteichus hindustanus* [381,386] (GB Nos. AB103327, AJ579650 and AJ810851). Acetyltransferases and phosphotransferases, which may be involved in the self-resistance, are present outside of the clusters. Furthermore, two transporter proteins are present (TobU and TobT; GB Nos. CAH18564, and CAH18551, respectively).

Neomycin (fradiomycin), paromomycin, and lividomycin belong to 4,5-disubstituted 2-deoxystreptamine-containing aminoglycoside antibiotics. Their activities are compromised by methylation of 16S rRNA. The neomycin biosynthetic gene cluster was cloned from *Streptomyces fradiae* as a 50 kb DNA fragment (GB No. AJ629247). Two proteins, AphA [387] (GB No. CAF33306) and AacC8 (GB No. CAF33325), were proposed to be involved in the self-resistance. They are aminoglycoside 3′-phosphotransferase and aminoglycoside 3-acetyltransferase, respectively. In addition, two ABC transporters (GB Nos. CAF33314 and CAF33315) were detected within the cluster. The paromomycin biosynthetic gene cluster was cloned from *Streptomyces rimosus* as a 48 kb DNA fragment (GB No. AJ628955). The aminoglycoside 3′-phosphotransferase (ParR) and ABC transporters (ParT and ParU) that are located within the cluster are homologous to those in the neomycin biosynthetic gene cluster. Interestingly, two acetyltransferases (GB Nos. CAG44462 and CAG44463) that are involved in self-resistance are present in other locations [388] (GB No. AJ749845). The lividomycin biosynthetic gene cluster was cloned from *Streptomyces lividus* as a 40 kb DNA fragment (GB No. AJ748832). No resistance-related gene was detected within the cluster, except two ABC transporter genes (GB Nos. CAG38699 and CAG38700). Hygromycin B is an aminocyclitol antibiotic that inhibits the protein synthesis and 30S ribosomal subunit assembly. The producer *Streptomyces hygroscopicus* is highly resistant to hygromycin B due to the presence of hygromycin B phosphotransferase activity [389]. Hygromycin A, structurally unrelated to hygromycin B, inhibits the peptidyltranferase reaction of protein synthesis. The hygromycin A biosynthetic gene cluster of 31.5 kb DNA fragment was cloned [390]. *O*-phosphotransferase Hyg21 is involved in self-resistance [391]. Istamycin that is produced by *Streptomyces tenjimariensis* is an aminoglycoside antibiotic [392]. FmrT consisting of 211 amino acid residues was proposed to be the rRNA methyltransferase involved in self-resistance [393]. Istamycin is also acetylated by kasugamycin-producing *Streptomyces kasugaensis*. [394]. Kasugamycin is another aminoglycoside antibiotic that is produced by *S. kasugaensis*. It is used mainly for the prevention of the growth of a fungus causing rice blast disease. The ABC transporter genes, *kasKLM*, are responsible for the self-resistance of a kasugamycin-producer strain [395] (GB No. AB033992). Fortimicin (astromicin) is an aminoglycoside antibiotic that is produced by *Micromonospora olivasterospora*, and *fmrO* encoding 16S rRNA methyltransferase, plays a role in self-resistance [396]. The streptothricin group antibiotics show a broad antibacterial spectrum. However, their characteristic delayed toxicity prevents their clinical application. The biosynthetic gene cluster contains genes for an acetyltransferase and two ABC transporters, which may play a role in self-resistance [397,398,399] (GB Nos. AB684620 and AB684619).

The macrolide antibiotics are a class of natural products that consist of a large macrocyclic lactone ring, to which one or more deoxy sugars are attached [400,401]. The lactone rings are usually 14-, 15-, or 16-membered. Erythromycin and oleandomycin belong to 14-membered macrolides. The erythromycin biosynthetic gene clusters were cloned from *Saccharopolyspora erythraea* and *Actinopolyspora erythraea* [402,403,404] (GB No. AM420293). Within the clusters, *ermE* encoding *N*-6-aminoadenine-*N*-methyltransferase is involved in self-resistance. Esterases, efflux proteins, phosphotransferases, acetyltransferases, glycosyltransferases, and dioxygenases are also proposed to be involved in self-resistance [404]. Two ABC transporters (OleB and OleC) and a glycosyltransferase (OleD) are proposed to be involved in the self-resistance of oleandomycin-producing *Streptomyces antibioticus* [405,406]. Tylosin belongs to a 16-membered macrolide antibiotic. The tylosin biosynthetic gene cluster was cloned from *Streptomyces fradiae* [407]. The resistance genes encoding rRNA methyltransferase (*tlrB*; GB No. AAD12162) and the ABC transporter (*tlrC*; GB No. AAA26832) are located at both ends of the cluster. Mycinamicin, which is produced by *Micromonospora griseorubida*, is a 16-membered macrolide antibiotic. The biosynthetic gene cluster was cloned [408]. The myrB encoding rRNA, methyltransferase, is involved in self-resistance [409]. Methymycin is a 12-membered macrolide that is isolated from *Streptomyces venezuelae*. The modification of 23S rRNA by PikR1 and PikR2, and glycosylation/deglycosylation play the self-resistance in the producing strain [410].

Tetracyclines are members of the polyketide family natural products, and are characterized by their tetracyclic ring structure [411,412]. The gene cluster for oxytetracycline biosynthesis was cloned from *Streptomyces rimosus* as a 25 kb DNA fragment, including bacterial Type II polyketide synthases genes [413,414] (GB No. DQ143963). Two resistance-related genes, *otrA* and *otrB,* are present at both ends. The *otrA* encodes the TetM-like ribosome protection protein [415] (GB No. CAA37477) and the *otrB* encodes an efflux MFS transporter (GB No. AOR83343). The gene cluster for chlortetracycline biosynthesis was cloned from *Streptomyces* (*Kitasatospora*) *aureofaciens* (GB Nos. CP020567 and HM627755). Two resistance-related proteins (GB Nos. ARF80631 and ARF80644) are detected within the cluster. They are the MFS efflux transporter and the GTP-binding ribosomal protection protein [416,417], respectively. In addition, OtrC proteins (ABC transporters, GB Nos. AAR96051 and AAR96052) are suggested to be concerned with the self-resistance [418,419].

Chloramphenicol is an antibiotic that is produced by *Streptomyces venezuelae* and other *Streptomyces* species. It behaves primarily by inhibiting protein synthesis and is used for the treatment of Gram-positive and Gram-negative bacterial infections. However, the side effects, such as bone marrow suppression, nausea, and diarrhea, restrict its common use. The whole DNA sequence of the *S*. *venezuelae* genome was determined, including the chloramphenicol biosynthetic gene cluster [420] (GB Nos. FR845719 and AF262220). Two transporters [421] (GB Nos. CCA54203 and CCA57351), acetyltransferase [422], and a phosphotransferase [423,424] (GB No. CCA57350) were reported to be involved in self-resistance. In addition, the chloramphenicol hydrolase that removes the dichloroacetyl moiety from chloramphenicol may be involved in the resistance [425]. However, chloramphenicol acetyltransferase activities, which are responsible for the inactivation of chloramphenicol in various bacteria, including *Streptomyces coelicolor* Mueller, *S. acrimycini*, and *S. griseus*, are not detectable in chloramphenicol-producing *S. venezuelae* [426,427].

The glycopeptide antibiotic, vancomycin, and the structurally related antibiotics are supposed to be the last lines of defense against a variety of serious infections that are caused by Gram-positive bacteria. These antibiotics cannot penetrate the peptidoglycan layer and do not act against Gram-negative bacteria. The gene cluster for vancomycin biosynthesis was cloned from *Amycolatopsis orientalis* (GB No. HE589771). Within the cluster, the ABC transporter (GB No. CCD33134) is located. Besides this, VanHAX resistance cassette exist [428]. These enzymes participate in the resistance mechanism in vancomycin-resistant enterococci strains by redirecting a portion of the peptidoglycan pathway [429,430,431]. Interestingly, the VanHAX is detectable not only in vancomycin-related glycopeptide-producing *Actinoplanes teichomyceticus* [432] and *Streptomyces toyokaensis* [433], but also in non-producing *Streptomyces coelicolor* (GB No. AL939117). The biosynthetic gene clusters for vancomycin-related glycopeptide antibiotics, such as balhimycin, chloroeremomycin, A40926, A47934, and teicoplanin were cloned [432,433,434,435,436,437,438,439,440,441]. The ABC transporters are present in all of these clusters. It is reported that the glycopeptide antibiotic A40926 producer *Nonomuraea* species possesses a novel d,d-peptidase/d,d-carboxypeptidase, which is involved in self-resistance and peptidoglycan maturation [442]. Moenomycins, which are produced by *Streptomyces ghanaensis* and related organisms, are phosphoglycolipid antibiotics that target peptidoglycan glycosyltransferases that are involved in bacterial cell wall biosynthesis. Transporter proteins are present within the cluster [443,444]. Friulimicin is a lipopeptide antibiotic that is produced by *Actinoplanes friuliensis*. Transporter proteins within the cluster were reported to be involved in the self-resistance [445]. Bleomycin that is produced by *Streptomyces verticillus* is a DNA synthesis inhibitor and has been used for cancer chemotherapy. Two resistant determinants, *blmA* and *blmB*, were isolated encoding an acetyltransferase and the bleomycin-binding protein, respectively [446]. Zorbamycin is a member of the bleomycin family glycopeptide anti-tumor antibiotic. In contrast to those of bleomycin and tallysomycin, another bleomycin family antibiotic, zorbamycin producer *S. flavoviris*, lacks the *N*-acetyltransferase, and the zorbamycin-binding protein is sufficient to confer resistance in the producing bacteria [447]. 

β-Lactam antibiotics, including penicillins and cephalosporins, are the most commonly used antibiotics, although they have been used for almost one century. β-Lactam antibiotics are classified into five groups, namely: penicillins, cephalosporins/cephamycins, clavulanic acid, thienamycin, and nocardicin A and sulfazecin. Penicillin is the first antibiotic that has been isolated as a natural secondary metabolite [448]. Penicillins and cephalosporins/cephamycins are produced by bacteria, as well as fungi, while others are produced by bacteria [449,450]. The gene clusters for the biosyntheses of penicillins/cephalosporins/cephamycins were cloned from *Streptomyces clavuligerus* [451,452] (GB No. CM000913), *S. cattleya* [453] (GB No. FQ859185), *Nocardia lactamdurans* [454,455], *Lysobacter lactamgenus* [456] (GB No. X56660), *Penicillium chrysogenum* [457] (GB No. AM920436), and *Aspergillus nidulans* [458] (GB Nos. AH000059 and X54853). The genes for β-lactamases and penicillin-binding proteins, which are involved in the self-resistance in bacteria [459,460,461], are present within these clusters of bacteria, whereas they are absent in those of the fungi. Clavulanic acid is an inhibitor of various kinds of β-lactamases from pathogenic bacteria and was isolated from *S. clavuligerus* [462]. It is used in combination with β-lactam antibiotics. The gene cluster for the biosynthesis of clavulanic acid is located between the cephamycin gene cluster and penicillin-binding protein, and β-lactamase genes [452,463,464]. A comparison of the cephamycin gene clusters of *S. clavuligerus* and *S. cattleya*, a clavulanic acid-non-producer, indicates that the clavulanic acid gene cluster is inserted between the cephamycin gene cluster and penicillin-binding protein/β-lactamase genes, without affecting the presence of the penicillin-binding protein and β-lactamase genes, suggesting that the penicillin-binding proteins and the β-lactamases play important roles in the protection from cephamycin, but not from clavulanic acid in the producer. However, the precise role of penicillin-binding protein and β-lactamase genes in clavulanic acid biosynthesis remains to be elucidated [465].

Thienamycin is the progenitor natural product of the broad-spectrum carbapenem antibiotics [466]. The gene cluster for the biosynthesis of thienamycin is located in the plasmid of *S. cattleya* [467,468] (GB No. AJ421798). There are three genes that are involved in self-resistance within the cluster, that is, *thnF*, *thnJ*, and *thnS*, by encoding *N*-acetyltransferase, transport protein, and β-lactamase, respectively [467]. In addition, the *thnC* encoding efflux pump may be implicated in the resistance. Nocardicin A is a monocyclic β-lactam antibiotic monobactam, and was isolated from *Nocardia uniformis* [469] and other actinomycetes. It shows moderate activity against Gram-negative bacteria and exhibits some β-lactamase resistance. The biosynthetic gene cluster of nocardicin A was cloned [470] (GB No. AY541063). Acetyltransferase (NocD) and the transporter protein (NocH) were proposed to be involved in self-resistance. In accord with the β-lactamase resistance, the β-lactamase gene was deficient. Another monobactam antibiotic sulfazecin was isolated from *Pseudomonas acidophila*. It is active against Gram-negative bacteria [471] and is not inactivated by metallo-β-lactamases, which renders bacteria with extended-spectrum β-lactam resistance. The gene cluster contains several transporter genes, a β-lactamase gene, and the multidrug transporter gene *mdtB*, which may be involved in the self-resistance [472] (GB No. KX757706). The exact role of β-lactamase remains to be clarified.

Viomycin is a member of the tuberactinomycin family of antibiotics, which are peptide antibiotics containing nonprotenogenic amino acids. They are essential drugs against *Mycobacterium tuberculosis*. The viomycin biosynthetic gene cluster was cloned and sequenced. The *vph* gene encoding viomycin phosphotransferase is involved in the self-resistance [473]. Pristinamycin that is produced by *Streptomyces pristinaespiralis* is a streptogramin group antibiotic. The DNA fragment of 120 kb covers the pristinamycin-specific genes for the biosynthesis, regulation, and resistance of pristinamycin, although multidrug resistance gene *ptr* is located outside the 210 kb supercluster [474,475]. Lincomycin and celesticetin are lincosamide antibiotics. Resistance is usually encountered in the form of the MLS phenotype, which includes macrolides, lincosamides, and streptogramin B type antibiotics [476]. The *clr* gene product, rRNA methyltransferase, is involved in self-resistance [477]. Daunorubisin and doxorubin are clinically important anthracycline antitumor antibiotics that are isolated from *Streptomyces peucetius.* Four proteins were reported to be involved in self-resistance. DrrA and DrrB proteins form an ATP cassette transporter/antiporter system [478]. The DrrC protein is a DNA-binding protein, like an UvrA-like protein [479]. The DrrD protein may function as oxygen oxidoreductase, like McrA in mitomycin C resistance [480]. Chromomycin A_3_ is an aureolic acid group antitumor antibiotic that is produced by *Streptomyces griseus*. The biosynthetic gene cluster contains three genes that are involved in the self-resistance. The *cmrA* and *cmrB* genes encode the ABC transporters, and *cmrX* encodes a UvrA-like protein of UV repair nuclease [481]. Mithramycin is another aureolic acid type antitumor antibiotic that is produced by the *Streptomyces* species [482]. The biosynthetic gene cluster of mithramycin was cloned [483] (GB No. X89899). The three genes (*mtrX*, *mtrA* and *mtrB*) that are located at the end of the cluster were proposed to be involved in the self-resistance, two of which encode the ABC transporters. Novobiocin is a member of the aminocoumarin type of antibiotics, which include coumermycin A_1_ and clorobiocin. The novobiocin biosynthetic gene cluster contains two self-resistance genes, *novA* and *gyrB*. The former encodes the ABC transporter and the latter encodes novobiocin-resistant gyrase subunit B [484] (GB No. AF170880). The biosynthetic gene clusters of coumermycin A_1_ and clorobiocin were also cloned and sequenced [485,486]. Mitomycin C is an antitumor antibiotic that is produced by *Streptomyces lavendulae*. Two self-resistance proteins were reported, one is oxygen oxidoreductase (Mcr) and the other is mitomycin-binding protein (Mrd) [487,488]. Yatakemycin is an antitumor antibiotic belonging to the family of CC-1065, and duocarmycin, which are produced by *Streptomyces* species. They are DNA-alkylating agents. The biosynthetic gene cluster contains five self-resistance-related genes including DNA glycosylase, DNase, and the transporter [489,490]. Natamycin is a polyene macrolide antifungal antibiotic. ABC transporters are involved in self-resistance [491]. Capuramycin is a nucleoside antibiotic that is isolated from the *Amycolatopsis* species, which inhibits bacterial translocase I that is involved in peptidoglycan cell wall biosynthesis. The gene cluster for the biosynthesis of capuramycin was cloned [492] (GB No. KP995196). The phosphotransferase gene *capP*, located at the end of the cluster, is implicated in the self-resistance. A-500359s are nucleoside antibiotics inhibiting phosphor-*N*-acetylmuramyl-pentapeptide translocase. A phospho-transferase is involved in the self-resistance [493]. Laspartomycin is a lipopeptide antibiotic that is produced by *Streptomyces viridochromogenes* [494]. The gene cluster was cloned [495]. Three transporters were proposed to be involved in the self-resistance. Platensimycin (PTM) and platencin (PTN) are bacterial fatty acid synthase inhibitors that are produced by *Streptomyces platensis*. PtmP3/PtnP3 and FabF proteins confer PTM and PTN self-resistance by target replacement and target modification [496]. d-Cycloserine is an anti-tubercular antibiotic that is produced by *Streptomyces lavendulae*. The biosynthetic gene cluster was cloned [497]. The d-alanyl-d-alanine ligase DcsI and a membrane protein, DcsJ, are involved in self-resistance. Fosfomycin has a unique chemical structure, containing a carbon-phosphorus and an epoxide. It inhibits peptidoglycan biosynthesis. The FomA and FomB proteins confer self-resistance on the producer organism by the phosphorylation of fosfomycin and fosfomycin monophosphate, respectively [498]. The rifamycins are broad-spectrum antibiotics that inhibit bacterial RNA polymerase. Several self-resistance mechanisms have been reported, including RNA polymerase modification, glycosylation, phosphorylation, and transporters [499,500]. Thiostrepton, micrococcin, nosiheptide, promothiocin, and promoinducin are ribosomally produced thiopeptide antibiotics that are produced by the *Streptomyces*, *Bacillus*, and *Micrococcus* species. The mutation of the ribosomal protein L11 is the mechanism of self-resistance [501], and rRNA methylation may be also involved [502]. 

Neocarzinostatin is an enediyne type antitumor antibiotic that is synthesized by *Streptomyces carzinostaticus* in the form of a chromoprotein complex. The amino acid residues D33 and D99 of the apoprotein play significant roles for self-protection. In addition, the neocarzinostatn carrier protein and mycothiol-dependent cellular detoxication are also important [503]. Calicheamicin is the non-chromoprotein enediyne type of antitumor antibiotic that is produced by *Micromonospora echinospora*. The biosynthetic gene cluster was cloned [504]. The non-heme iron metalloprotein CalC within the cluster is participated in the self-resistance [505] (GB No. AF497482). CalU16 and CalU19 are reported to be structural homologues of CalC. Kedarcidin is another enediyne type chromoprotein antitumor antibiotic that is isolated from the *Streptoalloteichus* species [506]. The *kedA*, *kedX*, and *kedX2* genes are involved in self-resistance, encoding apoprotein, efflux pump, and efflux pump, respectively [507]. Cyanosporasides, sporolides, and fijiolides are postulated to represent spontaneous enediyne degradation products. Cyanosporasides were isolated from the marine actinomycetes, *Salinispora pacifica* and *Streptomyces,* species. The biosynthetic gene clusters were cloned. It contains a couple of transporters and resistance proteins [508]. Salinosporamide A is a proteasome inhibitor that is isolated from the marine bacterium, *Salinispora tropica*. It shows anti-leukemic activity. The mutation of the 20S proteasome β-subunit confers self-resistance on the producer bacterium [509] (GB Nos. CP000667; EF397502). Miklamicin is a spirotetronate type of antibiotic that is produced by the endophytic *Micromonospora* sp. Three transporter proteins are present within the biosynthetic gene cluster [510] (GB No. LC021382). Microbisporicin is a lantibiotic antibiotic that is produced by *Microbispora coralline*. The biosynthetic gene cluster was cloned, which contains a couple of transporter proteins [511] (GB No. HM536998).

The self-resistance genes are also detected in Gram-positive *Bacillus* and the related species producing surfactin [512,513], subtilin [514], sublancin [515], zwittermicin [516,517], bacitracin [518], polymyxin [519] (GB No. EU371992), and edeine [520] (GB No. KC771276), and Gram-negative *Alcaligenes*, *Pseudomonas*, and fish pathogen *Yersinia* species, producing kalimantacin [521,522,523] (GB No. GU479979), pseudomonic acid [524], and holomycin [525], respectively. Holomycin is also produced by *Streptomyces clavuligerus* [526,527]. Interestingly, the resistance mechanisms are different between the Gram-negative *Yersinia* species and the Gram-positive *Streptomyces* species [525,527]. The biocontrol *Agrobacterium radiobacter* K84 secretes the antibiotic 84 that is selectively transported into the plant pathogen, *A. tumefaciens*. The mutation of the leucyl-tRNA synthetase (LeuRS) is responsible for self-resistance [528]. The old antifungal antibiotic griseofulvin and an immunosuppressant drug, mycophenolic acid, were isolated from the *Penicillium* species. The transporter and IMP dehydrogenase are involved in self-resistance [529,530,531] (GB No. HQ731031). From these results, it is concluded that the antibiotic producers use more sophisticated resistance mechanisms than other organisms, such as rRNA methylation (modification of target), modification and detoxication of antibiotics by acetylation; phosphorylation and adenylylation; chemical degradation by, for example, β-lactamase; excretion of antibiotics by efflux pump; modification of target (e.g., tRNA synthetase); and so on. Table 5 shows the antibiotic resistance-related strategies in bacteria and fungi. The early stage and more recent researches on the antibiotic self-resistance were reviewed by Demain [532], Vining [533], Cundliffe [534], and Cundliffe and Demain [535].

## 8. Conclusions

Drug resistance, especially antibiotic resistance, is getting worse and worse. Antibiotic resistance genes (ARGs) are complex mixtures of the genes of intrinsic antibiotic resistance [536,537] and acquired resistance [538], and constitute resistome [369,370,371,372]. Resistome is composed of the ARGs from antibiotic producers and pathogenic bacteria, and cryptic genes and precursor genes. These genes interconnect with each other very closely by horizontal gene transfer and adaptive mutations. Moreover, resistome is now identified in almost every microbial community, including soil, activated sludge, human gut and oral microbiomes, and animal gut microbiomes [539,540]. On the other hand, it is now extremely difficult to find an effective antibiotic against resistant pathogenic bacteria, although every effort has been taken to discover new antibiotics [541,542,543]. One of the steps to solve this problem is to know precisely the mechanisms of drug resistance in the various kingdoms once more. This review compares the molecular mechanisms underpinning the self-resistance against phycotoxins, toxins from marine and terrestrial animals, plants, and fungi and antibiotics. The results show that each kingdom possesses the characteristic features, as follows: transporters/efflux pumps in phycotoxins, mutation of targets and sequestration in marine and terrestrial animal toxins, ABC transporters and sequestration in plant toxins, transporters in fungal toxins, and various mechanism in antibiotics, indicating that antibiotic producers make tremendous efforts for avoiding suicide by using an enormous array of strategies and are more flexible and adaptable to the changes of environments (Table 1, Table 2, Table 3, Table 4 and Table 5).

Self-resistance or self-defense is one of the conclusions after the long history of evolution and adaptation to the environment, where desperate struggles were experienced between predators and prey species for survival. The typical example is seen in tetrodotoxin. It is distributed in taxonomically diverse groups, from bacteria such as actinobacteria, bacteroides, firmicutes, and proteobacteria [140,141], to pufferfish, snakes, newts, and other animals [9,73]. Although the self-resistance mechanism to tetrodotoxin has not been explored in bacteriawhich, in animals, is as a result of the mutation of only a few restricted residues of the target, Nav (Figure 1). Similarly, the self-resistance mechanisms to toxins in plants are also limited to a few numbers, that is, sequestration and the related transporters. On the other hand, the mechanisms of antibiotic resistance are tremendously complex (Table 5). Antibiotics are thought to be toxins for pathogenic bacteria. For their survival, it is necessary for the pathogenic bacteria to behave as defensive prey against natural secondary metabolites, including antibiotics, as well as synthetic chemicals. Furthermore, compared to animals and plants, bacteria are more flexible to genetic variations, as described above. So it is extremely challenging to manage and overcome each of the mechanisms of the antibiotic resistance.

**Figure 6:**
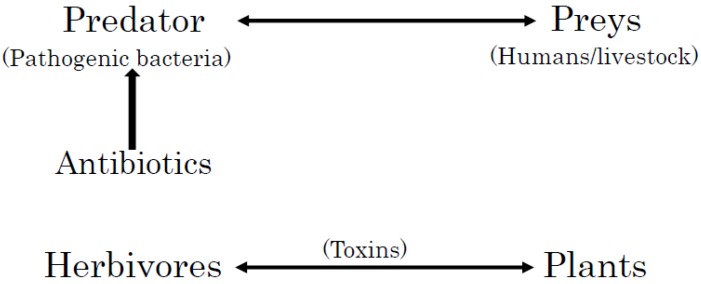
The relationship between predators and prey, and herbivores and plant.

The relation between pathogenic bacteria and humans/livestock is able to speak, figuratively, as that between predators and prey, or herbivores and plants (Figure 6). Arbuckle et al. described three types of gene-product based resistance, namely, toxin scavenging, target-site insensitivity, and off-target repurposing [9]. Immunological molecules correspond to toxin scavenging, and the pseudo-receptors to pathogenic bacteria play similar roles in target-site insensitivity, and the receptors with higher affinity to pathogenic bacteria, but no signaling ability, are candidates as off-target reprograming. These molecules, if possible, may act as significant functions for overcoming the drug resistance. As for the relationship between herbivores and plants, adaptation and sequestration in specific organs can be considered. To attain the adaptability to pathogenic bacteria in humans/livestock, some immunological activation is needed. Sequestration in specific organs of toxins, their non-toxic precursors, and enzymes to activate precursors, may be a dream, but I would expect these situations, considering recent technological developments.

With these features in mind, potential alternative strategies to overcome these resistance mechanisms are investigated in the following paragraph. Firstly, improvement of the directions of antibiotic usage. It is necessary for physicians to use narrow-spectrum, but not broad-spectrum, appropriate antibiotics in the right amounts, at the right time, and to identify the viral or bacterial pathogen precisely in a strain level, but not in the species level. Secondly, the innovation not to disseminate the resistance bacteria and resistance genes. Antibiotics are able to be prescribed coupled with bacteriophage [544,545], monoclonal antibody [546,547], and vaccines [548,549], or to be replaced with these approaches, if possible. Toxins are pivotal for preventing bacterial infection, when the activity of antitoxin is properly controlled [550,551]. The innate immune system, consisting of the immediate activation of the pathogen non-specific innate immunity, and following the activation of adaptive immune responses, is the last line of defense against the infectious diseases [552,553]. Lastly, the exploration of new compounds. Transporters or efflux pumps are the most fundamental and prevailing strategies for self-resistance and self-defense in every kingdom. So, inhibitors of transporters/efflux pumps are hopeful candidates for the prevention of infectious diseases. Together with the activation of silent genes, the combinatorial application of synthetic biological technology with genomic, metagenomics, and functional analyses of marine and terrestrial invertebrates, plants, and microbes will open the revival of the new golden era of natural products [554]. In fact, discoveries of teixobactin [555], compound 10 [556], and others [557], offer promising possibilities for a bright future. 

## Figures and Tables

**Table 1:** Resistance-related genes in biosynthetic gene clusters of phycotoxins and related compounds.

Toxin	Producing Species	GenBank Accession No. *^1^	Resistance-Related Gene/Protein	GenBank Accession No. *^2^	Reference
Cylindrospermopsin	*Cylindrospermopsis raciborskii* AWT205	EU140798	Multi-drug exporter: CyrK	ABX60156 (465aa)	[48]
	*Aphanizomenon* sp. strain 10E6	GQ385961	Multi-drug exporter: CyrK	ADF88272 (479aa)	[49]
	*Oscillatoria* sp. strain PCC 6506	FJ418586	Multi-drug exporter: CyrK	ADI48264 (479aa)	[47]
Jamaicamide A	*Lyngbya majuscula*	AY522504	No resistance-related gene		[50]
Hectochlorin	*Lyngbya majuscula*	AY974560	No resistance-related gene		[53]
Curacin A	*Lyngbya majuscula* strain 19L	AY652953	No resistance-related gene		[54]
	*Moorea producens* 3L	HQ696500	No resistance-related gene		[55]
Apratoxin A	*Lyngbya* (*Moorea*) *bouillonii* PNG5-198	MKZS01000001	No resistance-related gene		[57,58]
Lyngbyatoxin	*Lyngbya majuscula*	AY588942	No resistance-related gene		[59,60,61]
Teleocidin B	*Streptomyces blastmyceticus* NBRC 12747	AB937114	ABC transporters: Orf1, Orf2, Orf3	BAP27936-BAP27938	[62]
Microcystins	*Microcystis aeruginosa* PCC 7806	AF183408	ABC transporter: McyH	AAF00956 (538aa)	[65,66]
			Mutation of target: phosphatase?		[68]
Nodularin	*Nodularia spumigena*	AY210783	ABC transporter: NdaI	AAO64410 (601aa)	[64]
Saxitoxins	*Cylindrospermopsis raciborskii* T3	DQ787200	MATE: SxtF MATE: SxtM	ABI75096(471aa) ABI75103 (482aa)	[74,75]
	*Anabaena circinalis* AWQC131C	DQ787201	MATE: SxtM	ABI75138 (485aa)	[75]
	*Aphanizomenon* sp. NH5	EU603710	MATE: SxtM	ACG63815 (485aa)	[75]
	*Lyngbya wollei*	EU603711	MATE: SxtM1 MATE: SxtM2 MATE: SxtM3	ACG63829 (479aa) ACG63832 (485aa) ACZ26231 (503aa)	[76]
	*Raphidiopsis brookii* D9	ACYB00000000	MATE: SxtF (CRD_02147) MATE: SxtM (CRD_02155)	WP_009343300 (471aa) WP_040553734 (475aa)	[77]
	(*Mya arenaria*)		Mutation of target: sodium channel	AAX14719 (1435aa, partial)	[72]
Anatoxin A	*Oscillatoria* sp. PCC6506	FJ477836	AnaI: MATE-like transporter	AMO66168 (466aa)	[88,89]
	*Anabaena flos-aquae* 37	JF803645	No resistance-related gene		[90]
Hapalindole	*Fischerella* sp. ATCC 43239	KJ742064	No resistance-related gene		[96]
	*Fischerella* sp. PCC 9339		ABC transporter: Orf2 (277aa) ABC transporter: Orf3 (298aa) ABC transporter: Orf4 (331aa)	IMG Gene ID: 2517064626 IMG Gene ID: 2517064627 IMG Gene ID: 2517064628	[96]
Ambiguine	*Fischerella ambigua* UTEX 1903	KJ742065 KF664586	Efflux pump: AmbE1 Efflux pump: AmbE2 Efflux pump: AmbE3	AIJ28573 (388aa) AHB62754 (388aa) AIJ28574 (397aa) AHB62753 (397aa) AIJ28575 (151aa) AHB62752 (151aa)	[95,96]
Welwitindolinone	*Hapalosiphon welwitschii* UH IC-52-3	KJ767017	Multidrug resistance protein: WelE4	AIH14769 (105aa)	[96]
	*Hapalosiphon welwitschii* UTEX B1830	KF811479	No resistance-related gene		[94]
	*Westiella intricate* UH HT-29-1	KJ767018	Multidrug resistance protein: WelE4	AIH14815 (105aa)	[96]
Monensin	*Streptomyces cinnnamonensis*	AF440781	Efflux protein: MonT	ANZ52456	[103,104]
Palytoxin	*Palythora*, *Ostreopsis*, *Trichodesmium*		Sequestration?		[108]
Okadaic acid	*Protocentrum*		sulfated diesters?		[109]
Domoic acid	*Pseudo-nitzschia australis*		SLC6 transporter?		[116,117]
Patellamides	*Prochloron diene*	AY986476	No resistance-related gene		[123]
			ABC transporter?		[124]
Microcin B	*Pseudomonas antarctica* PAMC 27494	CP015600	ABC transporter: McbE ABC transporter: McbF	ANF87042 (237aa) ANF87073 (250aa)	[131]
Microcin C7	*Escherichia coli*	X57583	Efflux pump: MccC Acetyltransferase: MccE Self-immunity protein: MccF	CAA40810 (404aa) CAA40813 (521aa) CAA40814 (344aa)	[132,133,134]
Goadsporin	*Streptomyces* sp. TP-A0584	AB205012	ABC transporter: GodB ABC transporter: GodC Acetyltransferase: GodH	BAE46917 (550aa) BAE46948 (557aa) BAE46923 (222aa)	[129,135]

*^1^: GenBank accession number for the biosynthetic gene cluster of the toxin. *^2^: GenBank accession number for the resistance-related gene.

**Table 2:** Toxin resistance-related strategies in marine and terrestrial animals.

Toxin	Strategies	Animals	Reference
Tetrodotoxin	Mutation of target (Nav)	Fugu	[147,149]
		Newt	[144,145,148]
		Snake	[144,145,148]
Saxitoxin	Mutation of target (Nav)	Softshell clam	[72]
Conotoxin/Conopeptide	Mutation of target (Nav)	Conus	[154]
	Sequestration	Conus	[157,164]
	Post-traslational modification	Conus	[160,161,162,163]
Actinoporin	Modification of target (sphingomyelin)	Sea anemone	[168]
Hydralysin	Lack of receptor	Hydra	[169]
α-Bungarotoxin	Mutation and modification of target	Snake, mongoose, hedgehog, human	[180,181,182,183]
Atrolysins	Inhibition by serum	Opossum	[187]
		Rattlesnake	[188,189]
Botrocetin	Mutation of target (von Willebrand factor)	Opossum	[190]
Micrurotoxins	Mutation of target (GABA_A_ receptor)	Coral snake	[191]
LqTx (Scorpion α-toxin)	Mutation of target (Nav)	Scorpion	[199,200]
CssIV (Scorpion β-toxin)	Mutation of target (Nav)	Scorpion	[203,204]
Scorpion toxins	Mutation of target (Nav)?	Bat	[205,206]
Huwentoxin-IV (Tarantula toxin)	Mutation of target (Nav)	Tarantula	[215,216]
Jingzhaotoxin-I (Tarantula toxin)	Mutation of target (Nav)	Human	[218,219]
Lipophilic alkaloids	Sequestration	Frog	[223]
Batrachotoxin	Mutation of target (Nav)	Frog	[225,226]

**Table 3:** Toxin resistance-related strategies in plants.

Toxin	Strategies	Plant/Animal	Reference
Camptothecin	Mutation of target (Topo I)	*Ophiorrhiza japonica*	[233,234]
Sclareol	ABC transporter (NpPDR1)	*Nicotiana plumbaginifolia*	[235,236,237]
Berberine (Benzylisoquinoline alkaloid)	Excretion, ABC transporter	*Thalictrum minus*	[243]
	Sequestration, ABC transporter (CjABCB1)	*Coptis japonica*	[242,244]
Catharanthine (Indole alkaloid)	ABC transporter (CrTPT2)	*Catharanthus roseus*	[248]
Flavonoids	Sequestration, ABC transporter	*Arabidopsis*	[241,250]
Sanguinarine (Benzophenanthridine alkaloid)	Sequestration	*Papaver somniferum*	[252,253]
	Chemical modification	*Eschscholzia californica*	[254,255,256]
Coniine (Piperidine alkaloid)	Detoxication by cytochrome P450	*Trichoplusia ni*	[259]
Pyrrolizidine alkaloids	Sequestration as N-oxides	*Senecio*	[264]
	Sequestration as N-oxides	*Utetheisa ornatrix*	[265,266]
Steroidal glycoalkaloids	Deglycosylation to aglycones	*Solanaceae*	[269,270,271,275]
	Deglycosylation to aglycones	*Fusarium oxysporum*, *Cladosporium fulvum*	[272,273]
Benzoxazinoids	Glycosylation	*Secale cereale*	[276]
Iridoid glucosides	Glycosylation	*Plantago lanceolata*	[277,278]
Cyanogenic glucosides	Glycosylation	*Prunus*	[279,280]
Glucosinolates	Glycosylation, Myrosinase	*Brassicaceae*	[278,281]
Cardenolides	High K^+^ concentration	*Lepidopteran*	[282]
	Sequestration	*Lepidopteran*	[283]
	Mutation of target (Na/K^+^-ATPase)	*Danaus plexippus*, *Oncopeltus fasciatus*	[284,285,286,287]
	Sequestration/Exclusion, ABC transporter	*Daphnis nerii*	[288]
Nicotine	Exclusion/Cytochrome P-450	*Manduca sexta*	[290]
	Sequestration	*Nicotiana*	[291]
	α-DIOXYGENASE1	*Nicotiana attenuata*/*Manduca sexta*	[296]
Menthol (Monoterpene)	Sequestration	*Mentha canadensis*	[298]
Quinolizidine alkaloids	Sequestration	*Lupinus*	[299,301]
	Sequestration	*Macrosiphum albifrons*	[301]

**Table 4:** Toxin resistance-related strategies in fungi.

Toxin	Strategies	Fungi/Plant	Reference
Aflatoxin	Transporter, Hydroxylation	*Aspergillus flavus*, *Aspergillus parasiticus*	[304,305,306,307]
Sterigmatocystin	Not defined	*Aspergillus nidulans*	[309,310]
Ochratoxins	Transporter	*Aspergillus*, *Penicillium*	[312,313]
Citrinin	Transporter	*Monascus aurantiacus*, *Monascus purpureus*	[317,318]
Deoxynivalenol/Trichothecene	Glutathione-S-transferase, ABC transporter	Barley (plant)	[324]
Trichothecenes	Transporter	*Fusarium graminearum*	[325]
Zearalenone/Trichothecene	Monocarboxylate transporter	*Fusarium graminearum*/*Gibberella zeae*	[329,330]
	Lactonohydrolase	*Clonostachys rosea*	[331,332]
Fumonisins	Transporter	*Fusarium verticillioides*	[333,334]
	Transporter	*Aspergillus niger*, *A. welwitschiae*	[335,336]
Penitrem A	Transporter	*Penicillium crustosum*, *P. simplicissimum*	[337]
Roquefortine C	Transporter	*Penicillium chrysogenum*	[339]
Aphidicolin	Transporter	*Cephalosporium aphidicola*	[340]
Sirodesmin PL	Transporter	*Leptosphaeris maculans*	[342,343]
Gliotoxin	Transporter, Reductase	*Aspergillus fumigatus*	[342,344,345]
Ergot alkaloids	Not defined	Family Clavicipitaceae	[347,348,349,350,351,354]
Loline	Not defined	*Neotyphodium*/*Epichloae*/*Endophyte*	[356,358,359,360]
Lolitrem B	Not defined	*Neotyphodium*/*Epichloae*/*Endophyte*	[363,364]
Peramine	Transporter	*Epichloae festucae*	[362]
Swainsonine	Transporter	Orders Hypocreales, Chaetothyriales and others	[365]

**Table 5:** Antibiotic resistance-related strategies in bacteria and fungi.

Antibiotic	Strategies	Bacteria/Fungi	Reference
Streptomycin	Phosphorylation, Transporter	*Streptomyces griseus*	[377,378,379]
Kanamycin	Acetylation, rRNA methylation, Transporter	*Streptomyces kanamyceticus*	[381,382,383,384]
Gentamicin	rRNA methylation, Transporter	*Micromonospora echinospora*	[381]
Tobramycin	Phosphorylation, Acetylation, Transporter	*Streptoalloteichus hindustanus*	[381,386]
Neomycin	Phosphorylation, Acetylation, Transporter	*Streptomyces fradiae*	[387]
Paromomycin	Phosphorylation, Acetylation, Transporter	*Streptomyces rimosus*	[388]
Lividomycin	Transporter	*Streptomyces lividus*	AJ748832 *^1^
Hygromycin B	Phosphorylation	*Streptomyces hygroscopicus*	[389]
Hygromycin A	Phosphorylation, Transporter	*Streptomyces hygroscopicus*	[390,391]
Istamycin	rRNA methylation	*Streptomyces tenjimariensis*	[393]
Kasugamycin	Transporter, Acetylation	*Streptomyces kasugaensis*	[395]
Fortimicin/astromicin	rRNA methylation	*Micromonospora olivasterospora*	[396]
Streptothricin	Acetylation, Transporter	*Streptomyces lavendulae*	[397,398,399]
Erythromycin	rRNA methylation, Phosphorylation, Acetylation, Transporter	*Saccharopolyspora erythraea*	[402,403,404]
Oleandomycin	Glycosylation, Transporter	*Streptomyces antibioticus*	[405,406]
Tylosin	rRNA methylation, Transporter	*Streptomyces fradiae*	[407]
Mycinamicin	rRNA methylation	*Micromonospora griseorubida*	[408]
Methymycin	rRNA methylation, Glycosylation	*Streptomyces venezuelae*	[410]
Oxytetracycline	Ribosome protection, Transporter	*Streptomyces rimosus*	[413,414,415,417]
Chlortetracycline	Ribosome protection, Transporter	*Kitasatospora aureofaciens*	[416,417]
Chloramphenicol	Phosphorylation, Transporter, Acetylation, Hydrolase	*Streptomyces venezuelae*	[420,421,422,423,424]
Vancomycin	Transporter, Redirection of peptidoglycan biosynthesis	*Amycolatopsis orientalis*	[428,430], HE589771 *^1^
Balhimycin	Transporter, Redirection of peptidoglycan biosynthesis	*Amycolatopsis mediterranei*	[428,435,436]
Chloroeremomycin	Transporter	*Amycolatopsis orientalis*	[438]
Teicoplanin	Transporter, Redirection of peptidoglycan biosynthesis	*Actinoplanes teichomyceticus*	[432,434,437,439]
A40926	d,d-carboxypeptidase	*Nonomuraea* species	[440,441,442]
Moenomycin	Transporters	*Streptomyces ghanaensis*	[443,444]
Friulimicin	Transporters	*Actinoplanes friuliensis*	[445]
Bleomycin	Acetylation, Bleomycin-binding protein	*Streptomyces verticillus*	[446]
Zorbamycin	Zorbamycin-binding protein, Transporter	*Streptomyces flavoviridis*	[447]
Penicillin N/Cephamycin C	β-Lactamase, Penicillin-binding protein, Transporter	*Streptomyces clavuligerus*	[451,452]
	β-Lactamase, Penicillin-binding protein, Transporter	*Streptomyces cattleya*	[453]
	β-Lactamase, Penicillin-binding protein, Transporter	*Nocardia lactamdurans*	[454,455]
	β-Lactamase, Transporter	*Lysobacter lactamgenus*	[456], X56660 *^1^
Penicillin G	Transporter?	*Penicillium chrysogenum*	[457], AM920436 *^1^
	Transporter?	*Aspergillus nidulans*	[458], X54853 *^1^
Cephalosporin C	Unknown	*Acremonium chrysogenum*	AJ404737 *^1^
Clavulanic acid	β-Lactamase?, Transporter	*Streptomyces clavuligerus*	[451,459,460,463,464,465]
Thienamycin	β-Lactamase, Transporter, Acetylation	*Streptomyces cattleya*	[467,468]
Nocardicin A	Transporter, Acetylation	*Nocardia uniformis*	[470], AY541063 *^1^
Sulfazecin	β-Lactamase, Transporter	*Pseudomonas acidophila*	[472], KX757706 *^1^
Viomycin	Phosphorylation	*Streptomyces* species	[473], AY263398 *^1^
Pristinamycin	Transporter, Efflux pump	*Streptomyces pristinaespiralis*	[474,475]
Lincomycin	rRNA methylation	*Streptomyces caelestis*	[476,477]
Daunorubicin/Doxorubicin	Transporter, DNA-binding protein, Oxidoreductase?	*Streptomyces peucetius*	[478,479,480]
Chromomycin A_3_	Transporter, DNA-binding protein	*Streptomyces griseus*	[481]
Mithramycin	Transporter	*Streptomyces* species	[483], X89899 *^1^
Novobiocin	Transporter, Modification of target (gyrase)	*Streptomyces spheroides*	[484], AF170880 *^1^
Coumermycin A_1_	Transporter, Modification of target (gyrase, topoisomerase IV)	*Streptomyces rishiriensis*	[485]
Clorobiocin	Transporter, Modification of target (gyrase, topoisomerase IV)	*Streptomyces* species	[486]
Mitomycin	Oxidoreductases, Mitomycin-binding protein	*Streptomyces lavendulae*	[487,488]
Yatakemycin	Transporter, DNA glycosylase (DNA repair enzyme)	*Streptomyces* species	[489,490], JF429418 *^1^
Natamycin	Transporter	*Streptomyces chattanoogensis*	[491]
Capuramycin	Phosphorylation	*Amycolatopsis* species	[492], KP995196 *^1^
A-500359s	Phosphorylation	*Streptomyces griseus*	[493]
Laspartomycin	Transporters	*Streptomyces viridochromogenes*	[495]
Platensimycin/Platencin	Transporter, Target replacement/modification	*Streptomyces platensis*	[496]
d-Cycloserine	d-alanyl-d-alanine ligase, Membrane protein DcsJ	*Streptomyces lavendulae*	[497]
Fosfomycin	Phosphorylation	*Streptomyces wedmorensis*	[498]
Rifamycin	Transporter, Glycosylation, Phosphorylation, Target modification	*Nocardia* species	[499,500]
Thiopeptide antibiotics	Target modification (rRNA protein), rRNA methylation?	*Streptomyces azureus*	[501,502]
Neocarzinostatin	Modification of apo-protein, Sequestration, Mycothiol-dependent detoxication	*Streptomyces carzinostaticus*	[503]
Calicheamicin	Non-hem iron metalloprotein	*Micromonospora echinospora*	[504,505], AF497482 *^1^
Kedarcidin	Apoprotein, Transporters	*Streptoalloteichus* species	[506,507]
Cyanosporaside	Transporters	*Salinispora pacifica*, *Streptomyces* species	[508]
Salinosporamide A	Mutation of target (proteasome)	*Salinispora tropica*	[509], EF397502 *^1^
Maklamicin	Transporters	*Micromonospora* species	[510], LC021382 *^1^
Microbisporicin	Transporters	*Microbispora corallina*	[511], HM536998 *^1^
Surfactin	Transporters	*Bacillus subtilis*	[512,513]
Subtilin	Transporter	*Bacillus subtilis*	[514]
Sublancin	S-glycosylation	*Bacillus subtilis*	[515]
Zwittermicin	Transporters	*Bacillus cereus*, *Bacillus thuringiensis*	[516,517], HQ846969 *^1^
Bacitracin	Transporters	*Bacillus lichenifomis*	[518]
Polymyxin	Transporters	*Paenibacillus polymyxa*	[519], EU371992 *^1^
Edeine	Transporter, Acetylation	*Brevibacillus brevis*	[520], KC771276 *^1^
Kalimantacin	ACP reductase, Transporter	*Pseudomonas fluorescens*, *Alcaligenes* sp.	[522,523], GU479979 *^1^
Pseudomonic acid	tRNA synthetase	*Pseudomonas fluorescens*	[524]
Holomycin	rRNA methylation	*Yersinia ruckeri*	[525]
	S-methylation, Transporter	*Streptomyces clavuligerus*	[527], DS570652 *^1^
Agrocin 84	Mutation of target (tRNA synthetase)	*Agrobacterium radiobacter*	[528]
Griseofulvin	Transporter	*Penicillium* species	[529]
Mycophenolic acid	IMP dehydrogenase	*Penicillium* species	[530,531], HQ731031 *^1^

*^1^: GenBank accession number for the resistance-ralated gene.

## Founding

APC was sonspred by MDPI.
